# Doxorubicin-induced cardiotoxicity is suppressed by estrous-staged treatment and exogenous 17β-estradiol in female tumor-bearing spontaneously hypertensive rats

**DOI:** 10.1186/s13293-018-0183-9

**Published:** 2018-06-15

**Authors:** Kaytee L. Pokrzywinski, Thomas G. Biel, Elliot T. Rosen, Julia L. Bonanno, Baikuntha Aryal, Francesca Mascia, Delaram Moshkelani, Steven Mog, V. Ashutosh Rao

**Affiliations:** 1 Laboratory of Applied Biochemistry, Division of Biotechnology Review and Research III, Office of Biotechnology Research, Center for Drug Evaluation and Research, U.S. Food and Drug Administration, 10903 New Hampshire Ave., Bldg., Silver Spring, MD 20993 USA; 2 Division of Process Assessment III, Office of Process and Facilities, Office of Pharmaceutical Quality, Center for Drug Evaluation and Research, U.S. Food and Drug Administration, Silver Spring, MD USA; 3 Center for Food Safety and Applied Nutrition, U.S. Food and Drug Administration, College Park, MD USA

**Keywords:** Doxorubicin, Cardioprotection, Cardiomyopathy, Estradiol, Progesterone, Adriamycin

## Abstract

**Background:**

Doxorubicin (DOX), an anthracycline therapeutic, is widely used to treat a variety of cancer types and known to induce cardiomyopathy in a time and dose-dependent manner. Postmenopausal and hypertensive females are two high-risk groups for developing adverse effects following DOX treatment. This may suggest that endogenous reproductive hormones can in part suppress DOX-induced cardiotoxicity. Here, we investigated if the endogenous fluctuations in 17β-estradiol (E2) and progesterone (P4) can in part suppress DOX-induced cardiomyopathy in SST-2 tumor-bearing spontaneously hypersensitive rats (SHRs) and evaluate if exogenous administration of E2 and P4 can suppress DOX-induced cardiotoxicity in tumor-bearing ovariectomized SHRs (ovaSHRs).

**Methods:**

Vaginal cytology was performed on all animals to identify the stage of the estrous cycle. Estrous-staged SHRs received a single injection of saline, DOX, dexrazoxane (DRZ), or DOX combined with DRZ. OvaSHRs were implanted with time-releasing pellets that contained a carrier matrix (control), E2, P4, Tamoxifen (Tam), and combinations of E2 with P4 and Tam. Hormone pellet-implanted ovaSHRs received a single injection of saline or DOX. Cardiac troponin I (cTnI), E2, and P4 serum concentrations were measured before and after treatment in all animals. Cardiac damage and function were further assessed by echocardiography and histopathology. Weight, tumor size, and uterine width were measured for all animals.

**Results:**

In SHRs, estrous-staged DOX treatment altered acute estrous cycling that ultimately resulted in prolonged diestrus. Twelve days after DOX administration, all SHRs had comparable endogenous circulating E2. Thirteen days after DOX treatment, SHRs treated during proestrus had decreased cardiac output and increased cTnI as compared to animals treated during estrus and diestrus. DOX-induced tumor reduction was not affected by estrous-staged treatments. In ovaSHRs, exogenous administration of E2 suppressed DOX-induced cardiotoxicity, while P4-implanted ovaSHRs were partly resistant. However, ovaSHRs treated with E2 and P4 did not have cardioprotection against DOX-induced damage.

**Conclusions:**

This study demonstrates that estrous-staged treatments can alter the extent of cardiac damage caused by DOX in female SHRs. The study also supports that exogenous E2 can suppress DOX-induced myocardial damage in ovaSHRs.

**Electronic supplementary material:**

The online version of this article (10.1186/s13293-018-0183-9) contains supplementary material, which is available to authorized users.

## Background

Doxorubicin (DOX) is an anti-cancer drug in the anthracycline class used to treat a variety of malignances including breast tumors [1]. Doxorubicin causes cumulative cardiac toxicity, which limits the total life-time dose that can be safely administered [2, 3]. The onset of cardiotoxicity can occur during drug administration (acute), within a year following treatment (early onset), or years following therapy (late onset) [4, 5]. Doxorubicin-induced cardiac toxicity can be ameliorated to some extent by the concomitant use of the iron-chelating agent dexrazoxane (DRZ) [6]. The mechanism that DRZ uses to suppress myocardial damage remains controversial since reports demonstrate topoisomerase II inhibition and the suppression of iron-dependent ROS generation [7–10]. The lack of complete cardiac preservation supports a need to develop targeted, highly effective cardioprotectants. Additional insight into doxorubicin-induced cardiac toxicity may allow for the development of unique cardioprotective strategies.

Menopause and hypertension are two factors that could increase the risk of developing DOX-induced cardiomyopathy in patients [4, 11–14]. As previously described, the spontaneously hypertensive rat (SHR) model demonstrates good correlation between DOX-induced cardiotoxicity and troponin levels. The SHR model also provides low variability, uniform polygenic disposition, and a well-established biochemical response to anthracyclines [15, 16]. Similar to humans, SHRs have an increased incidence and severity of DOX-induced cardiotoxicity as compared to non-hypertensive Wistar rats [12]. Moreover, ovariectomizing female Wistar rats exacerbates DOX-induced cardiotoxicity [17]. In female SHR, ovariectomy did not increase cardiac damage as evinced by animals having similar serum cardiac troponin I levels [18]. Thus, the SST-2/SHR model is an established preclinically relevant and immuno-competent model to investigate the interactions between hypertension, immune responses, tumor growth, metastasis, and DOX-induced cardiomyopathy. Utilizing this preclinical model, experimental agents proposed to reduce DOX-induced cardiac damage can be further evaluated as potential therapeutics for hypertensive patients.

Clinical trials and animal studies have demonstrated that cardiac sensitivity to DOX is mediated by reproductive hormone levels [19]. Cardiac tissue is known to express progesterone (PgR) [20] and estrogen receptors (ERα, ERβ, and GPER), making the myocardium functionally responsive to endogenous and exogenous supplies of reproductive hormones [21]. Progesterone (P4) and 17β-estradiol (E2) are two steroid-reproductive hormones that can suppress in vitro DOX-induced cardiomyocyte oxidative damage in human cell lines [22–24]. Moreover, male and ovariectomized female Wistar rats subcutaneously injected with E2 suppress DOX-induced cardiotoxicity [17, 25, 26]. The efficacy of E2 to suppress DOX-induced cardiotoxicity in the presence of hypertension is unknown. In the absence of DOX, SHR-administered E2 did suppress endothelium-dependent coronary vascular dysfunction induced by oxidative damage [27]. This supports that SHR-administered E2 may suppress DOX-induced cardiotoxicity, but further investigation was required.

Female rats have a 4–5 day estrous cycle with fluctuations in the hormones E2 and P4 [28]. The circulating levels of E2 and P4 in rats can be used as a proxy to identify the estrous stage [29]. The four sequential stages of the estrous cycle are proestrus (PRO), estrus (EST), metestrus (MET), and diestrus (DIE). Proestrus has the highest circulating E2 and P4 levels which decline prior to estrus and remain low during metestrus, but gradually increase during diestrus [28]. Some degree of fluctuation can exist within each cycle [30–32]. Although several studies have focused on the effects of exogenous E2, there are no data regarding the effects of fluctuating endogenous hormones on DOX-induced cardiac toxicity in non-hypertensive and hypertensive rats.

In this report, we investigate if (1) endogenous E2 and P4 levels during estrous-staged DOX treatment can in part suppress cardiotoxicity in tumor-bearing adult female SHRs and (2) exogenous E2 and P4 can suppress DOX-induced cardiac damage in adult female ovariectomized spontaneous hypertensive rats (ovaSHRs). Collectively, our findings indicate DOX-treated rats during proestrus had an increase in cardiac damage and exogenous E2 protects against DOX-induced cardiomyopathy in ovaSHRs.

## Methods

### Chemicals and reagents

Twenty-one day time-release pellets were obtained from Innovative Research of America (Sarasota, FL). Pellets containing a carrier-binding matrix consisting of cholesterol, lactose, cellulose, phosphates, and stearates [vehicle (1.19 mg/day)], with the addition of either the hormones P4 (1.19 mg/day) and E2 (0.011 mg/day), alone and in combination (E2 + P4) or the chemotherapeutic tamoxifen free-base (0.14 mg/day) were subcutaneously implanted into the lateral side of the neck via trocar needle. The iron-chelator DRZ and chemotherapeutic agent DOX-HCl were obtained from Pfizer (New York, NY). A single DOX (10 mg/kg) treatment was administered via tail vein injection.

### Cell culture

SHR-derived breast cancer cells (SST-2) were obtained from Dr. Nozomu Koyangi (Eisai Co., Ltd. Clinical Research Center, Tokyo, Japan). SST-2 cells were cultured in RPMI 1640 media as described previously [18]. MCF-7 were obtained from ATCC and cultured in EMEM supplemented with 0.01 mg/ml bovine insulin and 10% FBS.

### Female spontaneously hypertensive rats

SHRs are a valid immune-competent and physiologically relevant rodent model [16, 18]. Female SHRs and ovaSHRs were purchased between 7 and 9 weeks of age from Envigo (Indianapolis, IN). Envigo ovariectomized SHRs between weeks 3 and 5 of age to generate ovaSHRs. All SHRs had a left femoral venous catheter (FVC), capped with an externalized PinPort™ (Instech, Plymouth Meeting, PA) and locked with a 50:50 heparin:glycerol solution (NC0373423; Thermo Fisher/SAI Infusion Technologies, Waltham, MA), allowing aseptic blood collection throughout the study. FVC surgeries were performed at Envigo.

Rats were housed individually in an environmentally controlled room (12-h light/dark cycle, 18–21 °C, 40–70% relative humidity) and provided food and water *ad libitum*. To avoid external estrogenic compounds, all SHRs were fed irradiated Teklad Global Soy Protein-Free Extruded Rodent Diet (2920x; Envigo). The Institutional Animal Care and Use Committee, Center for Drug Evaluation and Research, FDA approved the experimental protocol. The protocol was performed in an AAALAC-accredited facility. All procedures for animal care and housing complied with the Guide for the Care and Use of Laboratory Animals, 1996 (Institute of Laboratory Animal Research).

### SHR study design

SHRs were acclimated for 35 days to allow for proper estrous cycle staging (*n* = 100). After 35 days, 80 SHRs were cycling regularly and two consecutive cycles were obtained prior to starting the study. After which, animals were subcutaneously engrafted with 8.5 × 10^6^ SST-2 cells into the right mammary fat pad. This model results in development of a tumor at a rate of 100% [16, 18]. Twenty-four hours later, animals were divided to cohorts based on the estrous stage (proestrus, estrus, metestrus or diestrus) (*n* = 20 per stage) and treated once with injectable saline (0.9%) or DOX (10 mg/kg) via intravenous (IV) lateral tail vein injection, the cardioprotectant Dexrazoxane (DRZ; 50 mg/kg) via intraperitoneal injection (IP) or the combination of both DOX + DRZ (*n* = 5 per group). A graphical summary and list of all treatment groups, group sizes, and experimental procedures for the SHR study design can be found in Additional file 1: Figure S1 and Additional file 2: Table S1.

### OvaSHR study design

Ovariectomized SHRs (ovaSHRs) (*n* = 84) were acclimated for 5 days and then implanted with time-release pellets, containing a carrier matrix (vehicle) and either P4 (1.19 mg/day), E2 (0.011 mg/day), E2 + P4, Tam (0.14 mg/day), or Tam + E2 (*n* = 14 per group). The animals were acclimatized to the implants for 6 days. After which, the SHRs were subcutaneously implanted with of 8.5 × 10^6^ SST-2 cells into the right mammary fat pad. Twenty-four hours later, animals were treated once with injectable saline (0.9%) or DOX (10 mg/kg) via IV lateral tail vain injection (*n* = 6–7 per group). A graphical summary and list of all treatment groups, group sizes, and experimental procedures for the ovaSHR study design can be found in Additional file 1: Figure S2 and Additional file 2: Table S2.

### General health assessments

Body weight and tumor burden were measured every 2–4 days. Tumor volume was quantified using a caliper (after palpable) and calculated as previously described [33]. Blood collection was performed using FVC-PinPorts™ at the indicated days. SHRs and ovaSHRs were anesthetized and euthanized by exsanguination via the inferior vena cava. Heart and tumor tissues were immediately excised, divided, and flash frozen or fixed in a 10% neutral buffered formalin solution. At necropsy, images were acquired of the uterus from SHRs and ovaSHRs. The uterine width from estrous-staged SHRs that received saline (four groups; *n* = 4–5 per group), DOX (four groups, *n* = 4–5 per group), DRZ alone (four groups, *n* = 4–5), and DOX + DRZ (four groups, *n* = 4–5) were compared. The uterine width from vehicle- or DOX-treated ovaSHRs (two groups, *n* = 4–5 per group) was compared in addition to the effects of E2 (two groups, *n* = 5–6 per group) and P4 (two groups, *n* = 6 per group). The uterine width was quantitated using the open source software ImageJ [34] National Institutes of Health (Bethesda, MD) by taking averages of six cross sections of each uterus.

### Vaginal cytology

Vaginal cytology is a non-invasive method used to determine the stage of the estrous cycle based on the presence or absence of specific cell types and associated characteristics [35]. For example, proestrus contains nucleated epithelial cells, estrus lacks neutrophils but contains cornified cells, metestrus contains neutrophils with minimal cornified cells, and diestrus contains only neutrophils [35]. Therefore, in this study, vaginal lavage was used as a rapid assessment of the estrous cycle stage on all the SHRs and ovaSHRs. Vaginal lavage and cytology was performed with the SHRs for 2 weeks to confirm a continuous estrous cycle prior to any treatment. All SHRs that received saline, DOX, DRZ, and DOX + DRZ underwent daily vaginal lavage to assess the estrous cycle. All OvaSHRs underwent vaginal lavage for three consecutive days before pellet implantation and DOX administration in addition to the 5 days prior to necropsy. After collection, the slides were dried, fixed, and stained using the Dip Quick Stain Kit (NC9581034; Thermo Fisher Scientific) per the manufacturer’s instructions. Vaginal lavage was performed to minimize the un-wanted impacts on reproductive cycling as can be observed with other more invasive methods such as vaginal smears [35]. Slides were then imaged using a × 10 objective on a Panoramic MIDI digital slide scanner (3DHISTECH, Budapest, Hungary). Stages were classified based on the proportion of four cell types. The estrous stage was classified as previously described [36].

### Reproductive hormone levels and cardiac troponin I

Serum from the blood of estrous-staged SHRs (four groups) that received saline, DOX, DRZ, and DOX + DRZ (*n* = 3 per treatment per group) was collected at days 6 and 13 post treatment. Similar to SHRs, the serum was extracted from ovaSHRs implanted with pellets releasing a vehicle matrix (*n* = 14), E2 (*n* = 14), E2 + P4 (*n* = 14), E2+ Tam (*n* = 14), Tam (*n* = 14), and P4 (*n* = 14) at day 5 post pellet implantations. These pellet-implanted ovaSHRs were then divided to receive a vehicle control or DOX (two groups, *n* = 7 per treatment group), and at days 8 and 12 post treatment, blood was withdrawn to extract serum. Serum was separated from small (< 2 mL) blood samples in BD Microtainer® PST™ collection tubes containing lithium heparin (365985; Thermo Fisher Scientific). Serum was separated from large blood samples collected at necropsy with BD Vacutainer® SST™ collection tubes containing a silica clot activator (367988; Thermo Fisher Scientific). Serum hormone E2 was analyzed using the Mouse/Rat Estradiol ELISA kit (ES180S; CalbioTech, San Diego, CA) with an assay sensitivity between 3 and 300 pg/mL as determined by the manufacturer (*n* = 3–4 per treatment). Serum P4 was analyzed using the Mouse/Rat Progesterone ELISA kit (55-PROMS-E01; ALPCO, Salem, NH) with an assay sensitivity of 0.04 ng/mL (*n* = 3–4 per treatment). Both assays were performed on the Roche E Modular System (Roche Diagnostics, Indianapolis IN). Analyses were performed by CERLab at the Boston Children’s Hospital.

An aliquot of serum was used to evaluate levels of cardiac troponin I (cTnI) (*n* = 3–6 per treatment). The level of cTnI was measured using the SMC Human cTnI Immunoassay kit (03-0092-00; EMD Millipore, Darmstadt, Germany) on the Erenna platform according to the manufacturer’s instructions (Singulex, Alameda, CA). Three saline-treated animal samples were used to account for inter-plate variability.

### Histopathology

Formalin-fixed heart tissue was embedded in plastic and stained for H&E and toluidine blue within 2 weeks. Histopathology was performed by a board-certified pathologist. The frequency and severity of DOX-induced cardiac alterations (cardiomyopathy) was assessed by light microscopic examination of a cardiac section from each animal (*n* = 5–7 animals per group per treatment) according to the semi-quantitative method of Billingham [16, 18, 37]. This grading or scoring system is based on the percentage of cardiomyocytes showing myofibrillar loss and cytoplasmic vacuolization: 0 = no damage, 1≤ 5%, 1.5 = 5–15%, 2 = 16–25%, 2.5 = 26–35%, and 3 ≥ 35%.

### Echocardiogram

Echocardiogram measurements were performed with the Vevo2100 instrument, and data analysis was performed with the accompanying Vevo LAB software, v. 1.7.0 (VisualSonics, Toronto, Canada). M-mode interrogation was performed with the MS250 transducer (20 MHz) in the parasternal short-axis view. Measurements were performed in triplicate. All measurements were performed on lightly anesthetized animals while maintaining a heart rate of 350–400 beats per minute. The body temperature of the animal was maintained with a heated stage.

### Western blot

SST-2 and MCF-7 whole cell lysates were separated using SDS-PAGE and transferred to nitrocellulose membranes using a TransBlot® Turbo™ blotting system (Bio-Rad). Membranes underwent overnight incubation with the following antibodies: ER-alpha (Thermo Pierce, MA5-14501), ER-beta (Abcam, Ab-3576), PGRMC1 (Millipore, ABS776), and Her2 (Cell Signaling 2165) all diluted in 1:500 Odyssey LICOR blocking buffer (PBS). Immunoblots were imaged using an Odyssey Infrared Imager (LICOR).

### Statistical analyses

Statistical analyses were calculated using GraphPad Prism v6.05 (GraphPad, La Jolla, CA). When > 2 groups, with and without treatments were analyzed, a two-way analysis of variance (NOVA) with and without time as a repeated measure was performed with a Tukey’s post-test. An ANOVA was performed for > 2 untreated groups with and without time as a repeated measure followed by Tukey’s or Dunnett’s multiple comparison post-test. *p* values < 0.05 were considered statistically significant. Pearson’s correlations were used to analyze relationships between cTnI and hormones. A single outlier was excluded using a Grubbs test as previously described [18]. Detailed statistical analyses for each figure are listed in Additional file 2 with Tables S3–S6.

## Results

### DOX-induced estrous cycle irregularity in tumor-bearing SHRs

**Fig. 1:**
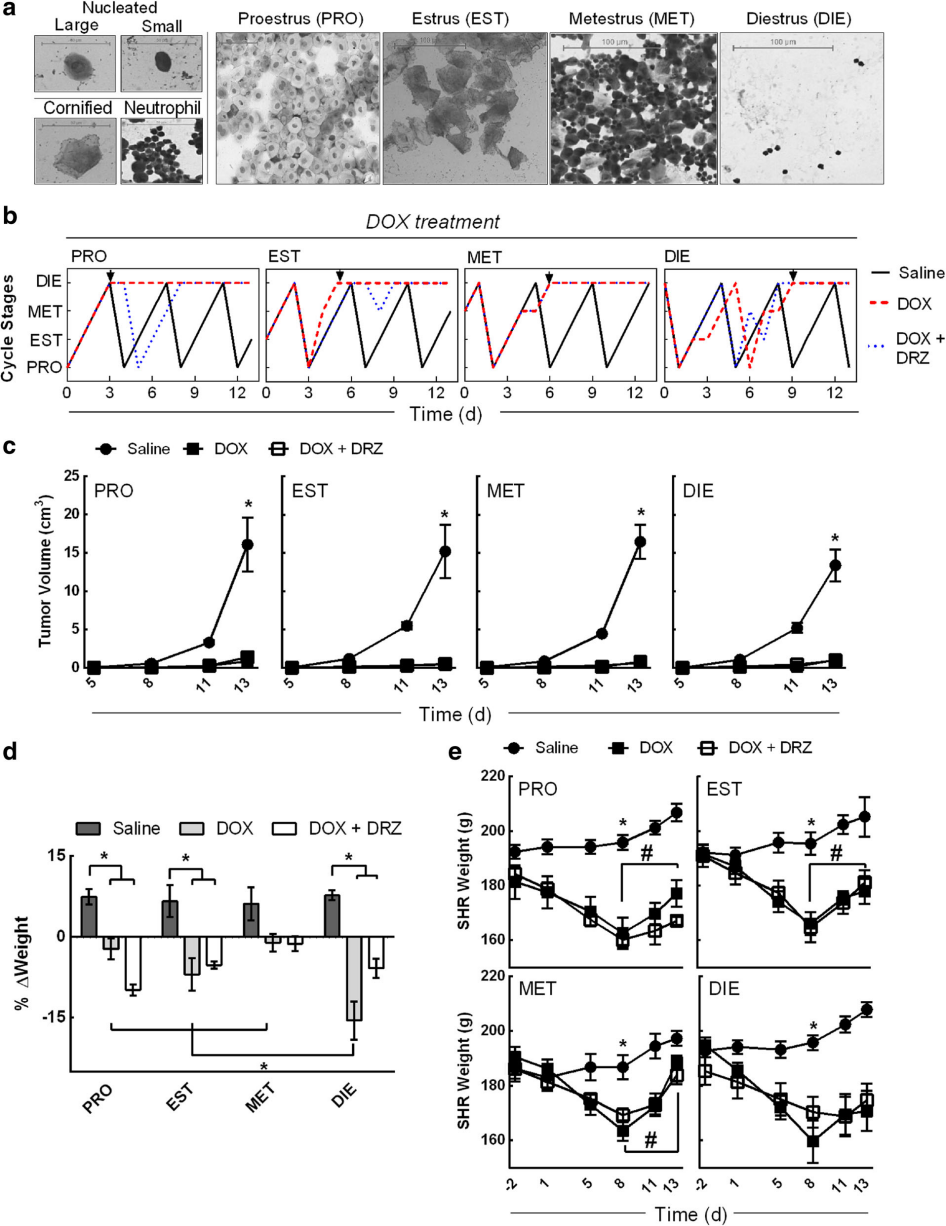
Estrous stage-specific DOX treatment causes cycle irregularity in tumor-bearing SHRs. **a** Representative vaginal cytologic images of nucleated, cornified, and neutrophil cells collected from SHRs during each stage of the estrous cycle: proestrus (PRO), estrus (EST), metestrus (MET), and diestrus (DIE). **b** Representative line graph depiction of the estrous cycle for 13 days following a single injection of saline, DOX, and DOX + DRZ during proestrus, estrus, metestrus, or diestrus. The estrous stages were determined by vaginal cytology in five animals per stage per treatment. Arrow indicates the onset of a prolonged diestrus phase. **c** SST-2 tumor volume in SHRs following a single treatment of saline, DOX, and DOX + DRZ during proestrus, estrus, metestrus, or diestrus at the indicated times. The data points represent the mean ± SEM. (two-way ANOVA performed on day 13, *n* = 4–5 animals per stage per treatment, *p < 0.05 according to Tukey’s multiple comparison between treatments within the stage.) **d** The SHRs weight change at day 13 after a single treatment during a specific estrous stage. Percentage of weight change was calculated by a day 13 to day 0 (no treatments) weight ratio. Bars represent the mean ± SEM. (Two-way ANOVA, *n* = 4–5 animals per treatment per stage, *p < 0.05 according to Tukey’s multiple comparison between stages and treatments.) **e** Weight in grams of SHR following DOX treatment during each estrous stage. (Repeated measure two-way ANOVA per stage, *n* = 4–5 animals per group per treatment, *p < 0.05 according to Tukey’s multiple comparison between treatments, while #*p* < 0.05 between the indicated days of the DOX and DOX + DRZ-treated groups.) Results of analyses are listed in Additional file 2: Table S3 and S4

Premenopausal women with early stage breast cancer that receive DOX undergo menstrual cycle irregularities leading to amenorrhea [38]. Similar to humans, female Wistar rats underwent estrous cycle irregularities following DOX treatment [39]. To investigate the effects of DOX on the estrous cycle in SHRs, vaginal cytologies were collected following the administration of saline, DOX or a combination of DOX and DRZ. Vaginal cytology is a non-invasive method used to determine the stage of the estrous cycle based on the absence and presence of specific cell types and associated characteristics [35]. Prior to any treatment, two consecutive 4-day estrous cycles were evaluated and used to establish the four animal cohorts, for subsequent DOX administration: proestrus, estrus, metestrus, and diestrus (Fig. 1a). Saline- and DRZ-treated SHRs maintained a continuous estrous cycle throughout the study (Fig. 1b and Additional file 1: Figure S3). In contrast to the saline injection, animals treated with DOX during proestrus, estrus, metestrus, or diestrus all underwent estrous cycle irregularity leading to prolonged diestrus (Fig. 1b). SHRs that received DOX during diestrus did not progress through one complete estrous cycle without exhibiting irregularity. SHRs treated with DOX during diestrus completed one 5-day estrous cycle (having prolonged estrus for 2 days). SHRs treated with DOX during diestrus also exhibited irregular cycling between days 5 and 9 at which point prolonged diestrus occurred for the duration of the study. The presence of DRZ did not prevent DOX-induced estrous cycle irregularity or prolonged diestrus for any treatment stage. Collectively, these data demonstrate that DOX can induce estrous cycle irregularity in tumor-bearing female SHRs.

To investigate if DOX treatment during a specific estrous stage altered the SHRs tumor burden and health, animals were monitored for changes in tumor volume, weight, and uterine width following a saline, DOX, or DOX + DRZ injection. DOX-treated SHRs during any of the estrous stages led to a decrease in tumor burden, weight gain, and uterine width as compared to the saline treatment (Fig. 1c, d and Additional file 1: Figure S4). At day 13 post SST-2 engraftment, significant tumors developed in all saline- or DRZ-treated SHRs (9.5 to 19.7 cm^3^; Fig. 1c and Additional file 1: Figure S5) as compared to DOX- and DOX + DRZ-treated SHRs (treatment effect, *p* < 0.0001). SHRs treated with DOX + DRZ during any of the estrous stages had a comparable reduction in the tumor burden as compared to the DOX treatment alone (0.4 to 1.2 cm^3^; Fig. 1c). This indicates the anti-cancer activity of DOX is independent of estrous stage in SHRs. The weight change from day 0 to day 13 was different in metestrus-staged SHRs treated with DOX and DOX + DRZ as compared to proestrus-, estrus-, and diestrus-staged animals, which decreased weight gain in the presence of DOX and DOX + DRZ (Fig. 1d) (interaction, *p* = 0.0034, stage and treatment). Diestrus-staged animals treated with DOX had the greatest weight loss as compared to the proestrus-, estrus-, and metestrus- staged animals treated with DOX. At necropsy, all saline- and DRZ-treated animals gained similar weight (3.8 to 8.0%) and estrous cycle stage had no impact on body weight changes associated with treatments (Fig. 1d and Additional file 1: Figure S5). Thirteen days after DOX administration, all SHRs that received DOX lost weight (− 1.1 to − 15.5%), but SHRs treated with DOX during diestrus lost the most amount of weight (− 15.5%) as compared to the DOX-treated SHRs during proestrus (− 2.3%), estrus (− 7.0%), and metestrus (1.1%). Following the changes in weight over time, all DOX-treated animals exhibited significant weight loss at day 8 post treatment, but only DOX- and DOX + DRZ-treated animals during diestrus did not regain weight between day 8 and day 12 (all stages, interaction, *p* ≤ 0.0003, time and treatment) (Fig. 1e).

Lastly, DOX has demonstrated to decrease uterine diameter in rodents [39, 40]. Therefore, uterine width was measured at necropsy. Treatment with DOX or DRZ + DOX coincided with a significant reduction in uterine width in all stages (0.16–0.17 cm) relative to the respective saline control (treatment effect, *p* < 0.0001). These data indicate that DOX treatment during any of the estrous stages causes a decreased the tumor growth, weight gain, and uterine width in SHRs.

### Estrous-staged treatment can alter the extent of DOX-induced cardiotoxicity

**Fig. 2:**
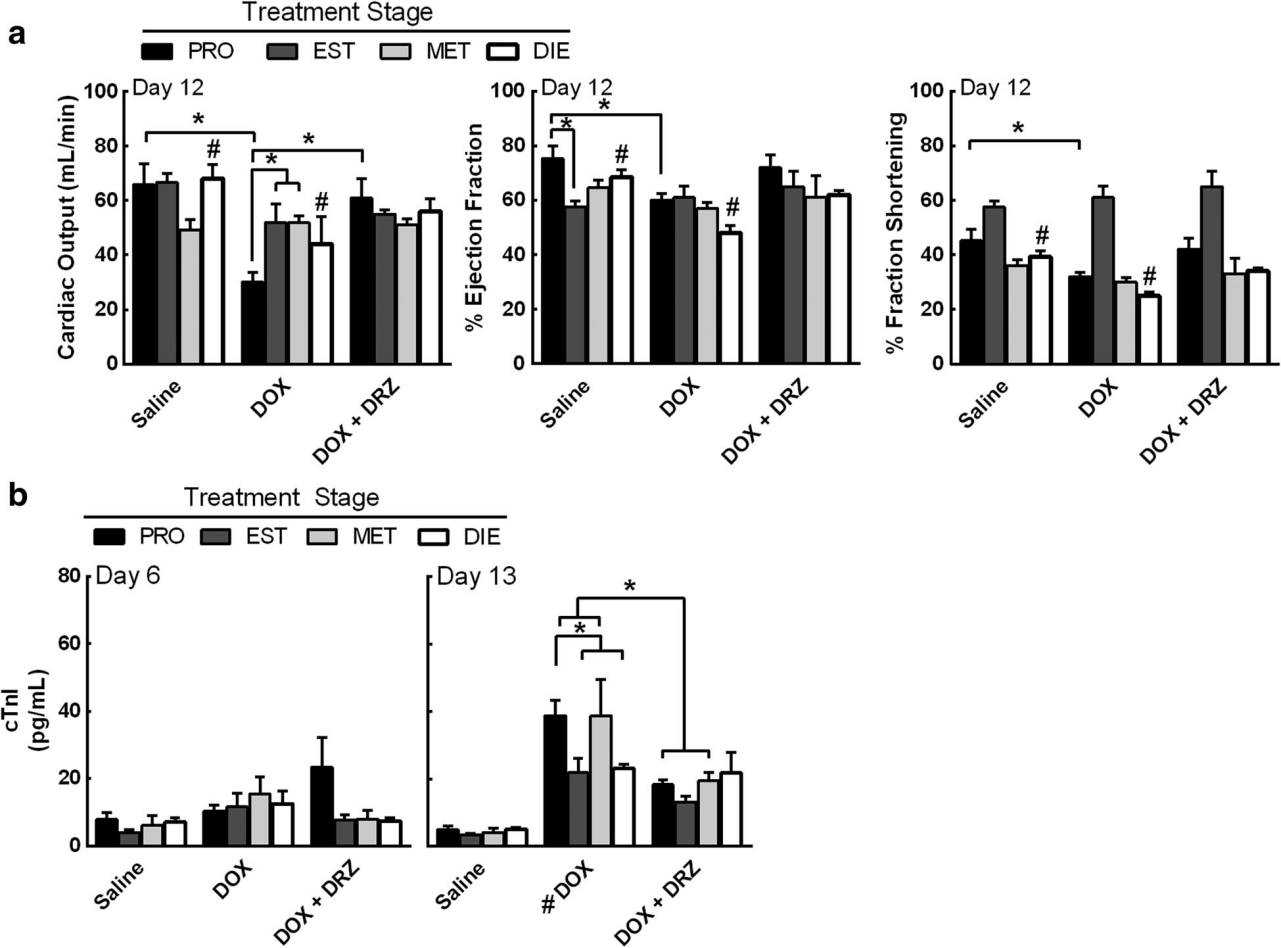
SHRs treated during proestrus are more sensitive to DOX-induced myocardial damage. **a** Echocardiograms were performed 12 days post treatment in estrous stage-specific SHRs. Cardiac output, % ejection fraction, and % fractional shortening were analyzed to assess myocardial dysfunction. **b** Serum concentrations of cardiac troponin I (cTnI) were measured from animals that received estrous stage specific treatments at day 13 post treatment. Bars represent the mean ± SEM. Two-way ANOVA results are displayed in Additional file 2: Table S3. *n* = 3 animals per treatment per stage. *p < 0.05 according to Tukey’s multiple comparison between stages and treatments, while #*p* < 0.05 between the indicated groups

To investigate if DOX treatment during a specific estrous stage can alter the extent of myocardial damage, cardiac dysfunction was measured 12 days after DOX treatment using echocardiography. Information from the echocardiograms was used to estimate cardiac output, left ventricular ejection fraction, and fractional shortening. There was no difference in cardiac output in animals treated with saline or DRZ during any estrous stage (Fig. 2a and Additional file 1: Figure S6). DOX-treated SHRs exhibited a proestrus and diestrus stage-dependent decline in cardiac output (interaction, *p* = 0.029, stages and treatments). The cardiac ejection fraction (stage effect, *p* = 0.034 and treatment effect, *p* = 0.004), and fractional shortening (stage effect, *p* < 0.0001 and treatment effect, *p* = 0.0097) did decrease in proestrus- and diestrus-staged SHRs that received DOX, but the stage-dependency was not established. There was limited to no recovery in cardiac functional tests when DOX-treated animals were supplemented with DRZ, except for proestrus-staged SHRs that received DOX + DRZ as compared to DOX alone (Fig. 2a). Animals that received DOX during proestrus had the greatest loss in cardiac output (30 mL/min) when compared to the SHRs that received DOX during estrus (52 mL/min), metestrus (52 mL/min), and diestrus (44 mL/min). Collectively, these data suggest that estrous-staged DOX treatment can alter the extent of myocardial damage and cardiac tissue may be more sensitive to DOX in SHRs during proestrus.

Cardiac troponin I (cTnI) is a specific cardiomyocyte protein, and serum cTnI levels are used as a method to assess myocardial damage [12, 18]. To verify DOX-induced myocardial damage in SHRs, the serum levels of cTnI were assessed from SHRs treated with DOX during proestrus, estrus, metestrus, or diestrus at day 6 and 13 post treatment (Fig. 2b). After 6 days, animals treated with DOX had a modest increase in cTnI values as compared to the saline control (treatment effect, *p* = 0.0008). No treatment caused the cTnI values to increase over 20 pg/mL on day 6. However, after 13 days, all estrous-staged animals that received DOX and DOX + DRZ had elevated cTnI levels as compared to animals injected with saline (treatment effect, *p* < 0.0001) (Fig. 2b). Proestrus- and metestrus-staged animals that received DOX showed 38–39 pg/mL of serum cTnI, which was higher than animals that received DOX during estrus (24 pg/mL) and diestrus (23 pg/mL), but the DOX-induced cTnI increase was not determined to be stage-dependent. Moreover, DRZ suppressed the elevation in serum cTnI in DOX-treated animals during proestrus (19 pg/mL) and metestrus (19 pg/mL).

To support the cTnI and echocardiography findings, histopathological analysis of cardiac tissue was performed (Table 1). Cardiomyopathy or lesion scores were assigned by the percentage of cardiomyocytes, within the sampled heart section that contain myofibrillar loss and cytoplasmic vacuolization as follows: no observed change (score 0), < 5% (score 1), 5–15% (score 1.5), 16–25% (score 2), 26–35% (score 2.5), and > 35% (score 3). All saline and DRZ controls displayed lesion scores (LS) less than 0.8, indicating less than 5% myofibrillar loss or cytoplasmic vacuolization within cardiomyocytes (Table 1). Each group that received DOX had a fraction of animals that exhibited an increase in microscopic cardiomyocyte damage. Diestrus-staged SHRs that received DOX had the highest levels of vacuolization and myofibrillar loss. Animals treated with DRZ alone had similar cTnI levels, histopathological scores, and cardiac function as the saline control (Additional file 1: Figure S6). These data suggest that DOX treatment during specific estrous stages may result in different degrees of cardiac toxicity.

**Table 1:** SHR cardiomyopathy score

Stage	Treatment	N	0	1	1.5	2	2.5	Average
PRO	Saline	5	5					0.0
	DOX	5	1	1	2	1		1.2
	DOX + DRZ	5	1	2	2			1.0
EST	Saline	5	1	4				0.8
	DOX	5	1	1	3			1.1
	DOX + DRZ	5	1	2	1	1		1.1
MET	Saline	5	2	3				0.6
	DOX	5	1	1	1	2		1.3
	DOX + DRZ	5	1	2	2			1.0
DIE	Saline	5	3	2				0.4
	DOX	5			1	2	2	2.1
	DOX + DRZ	5			4	1		1.6

Histopathology of cardiac sections from the SHRs at day 12 was assessed for cardiomyocyte lesions (myofibrillar loss and vacuolization) by a board-certified pathologist. The cardiomyopathy or lesion score indicates the percentage of affected cardiomyocytes within the sampled heart section: 0 is no observed change, 1 is < 5%, 1.5 is 5–15%, 2 is 16–25%, 2.5 is 26–35%, and 3 is > 35%

### Estrous-staged DOX treatment influences circulating 17β-estradiol and progesterone levels

**Fig. 3:**
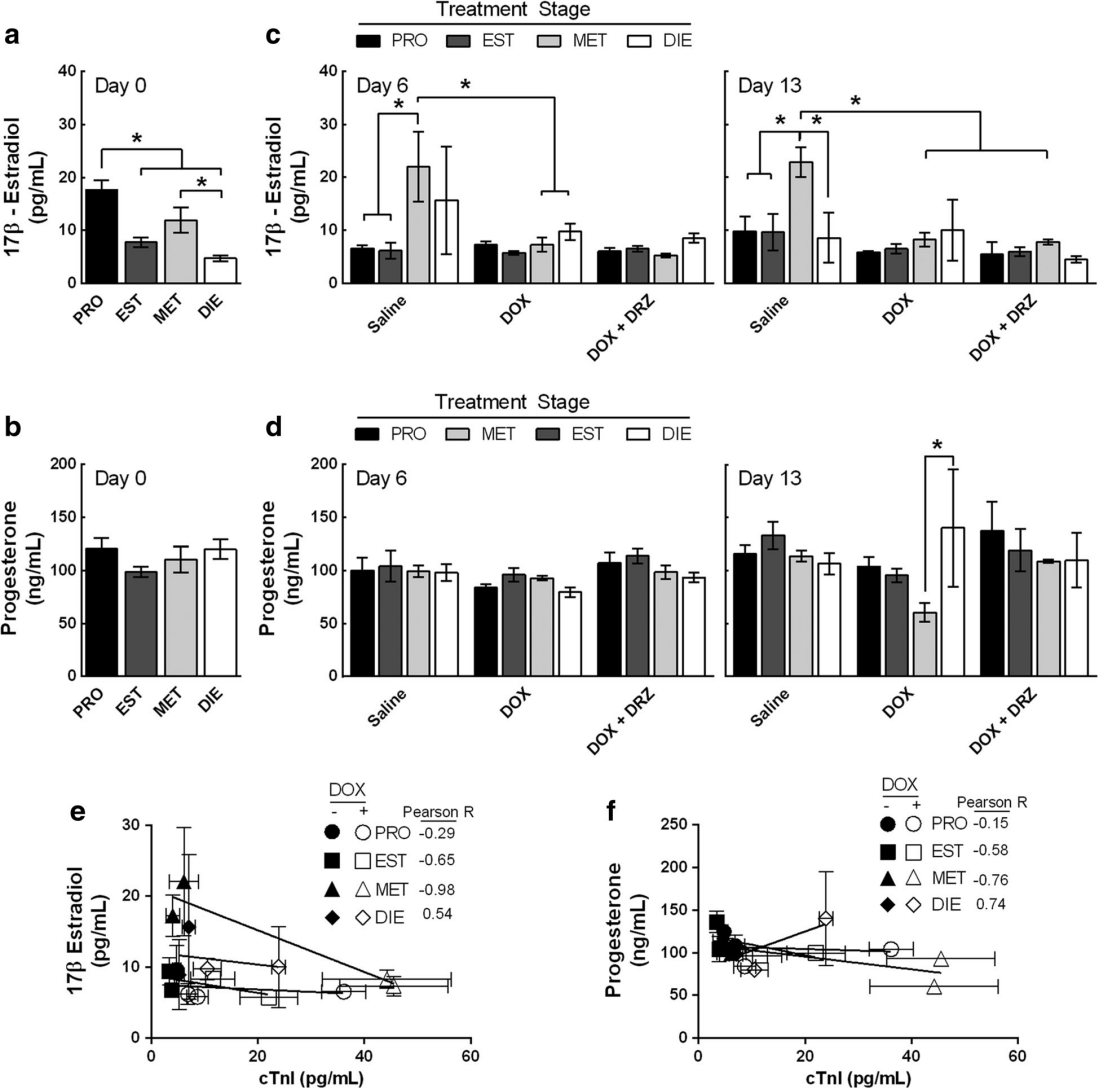
Cyclic regulation of the 17β-estradiol is lost in DOX-treated SHRs. **a**, **c** Serum concentrations of 17β-estradiol and progesterone were measured in estrous-staged SHRs at day 0 (no treatment) (ordinary ANOVA, *n* = 12 per stage, *p < 0.05 according to Tukey’s multiple comparison between stages). **b**, **d** 17β-estradiol and progesterone were measured in the presence of saline, DOX and DOX + DRZ at day 6 and 13 (Two-way ANOVA, *n* = 3–4 per treatment per stage, *p  <0.05 according to Tukey’s multiple comparison between treatments and stages). Bars represent the mean ± SEM. **e**, **f** Correlation between the levels of serum cardiac troponin I and serum 17β-estradiol or progesterone in SHRs. The data points represent the mean ± SEM

Estrogen and progesterone are two steroids that fluctuate during the estrous cycle [28]. Serum 17β-estradiol has been demonstrated to decrease in female SHRs following DOX treatment [18]. To investigate how the estrous cycle can affect serum concentrations of E2 and P4 following DOX treatment, serum from estrous-staged animals that received DOX was collected and analyzed to quantify E2 and P4 concentrations. Consistent with previous studies [28], the vaginal cytology-staged animals in proestrus animals had the highest levels of E2 (18 pg/mL) (*p* < 0.0001) (Fig. 3a). Moreover, saline-treated animals during metestrus were staged in proestrus using vaginal cytology and contained the highest E2 levels at day 6 (stage effects, *p* = 0.0194) and 13 (stage effects, *p* = 0.0438) (Figs. 3b and 1b). This indicates that saline-treated animals underwent normal estrous cycling and hormone fluctuations. In contrast to the saline-treated animals, DOX treatment to metestrus-staged animals suppressed the elevation in E2 to a concentration that was comparable between groups (5 to 8 pg/mL) at day 6 (interaction, *p* = 0.007, stages and treatments). However, prolonging the study to day 13 led to a loss in the stage-dependency of DOX-induced E2 depletion (stage effect, *p* < 0.0438 and treatment *p* = 0.0054). Prior to any treatments, the levels of P4 were comparable between all four estrous-staged animals (100 to 120 ng/mL) (Fig. 3c). The only observed difference was that metestrus-staged animals treated with DOX had a significant reduction in P4 after DOX treatment at day 13 as compared to diestrus-staged animals that received DOX (Fig. 3d). Proestrus- (104 ng/mL) and estrus- (95 ng/mL) staged animals treated with DOX did have greater circulating P4 than SHRs treated with DOX during metestrus (60 ng/mL). Collectively, these data demonstrate that DOX treatment decreases circulating E2 concentrations in SHRs, which may contribute to estrous cycle irregularity and cardiac dysfunction.

To examine for a potential relationship between E2 and P4 serum concentrations with DOX-induced myocardial damage in SHRs, the hormone concentrations were correlated with cTnI in animals that received DOX during a specific estrous stage. A moderate positive correlation (*R* = 0.54) was indicated between estrogen and cTnI when animals were treated during diestrus, which coincided with the lowest DOX-induced cardiotoxicity (Fig. 3e). Additionally, there was little correlation for SHRs treated in proestrus (*R* = − 0.29), a modest negative correlation for animals treated with DOX during estrus (*R* = − 0.65) and a strong negative correlation for animals treated in metestrus (*R* = − 0.98) (Fig. 3f). However, all estrous-staged animals that received DOX had low circulating E2 after 13 days. As for P4 and cTnI, a modest correlation was observed in diestrus-, estrus-, and proestrus- staged SHRs. Collectively, these data support that a single DOX treatment impairs E2 fluctuations during the estrous cycle, which may have led to elevated myocardial damage in SHRs.

### DOX influences 17β-estradiol mediated estrous cycle stimulation in ovaSHRs

**Fig. 4:**
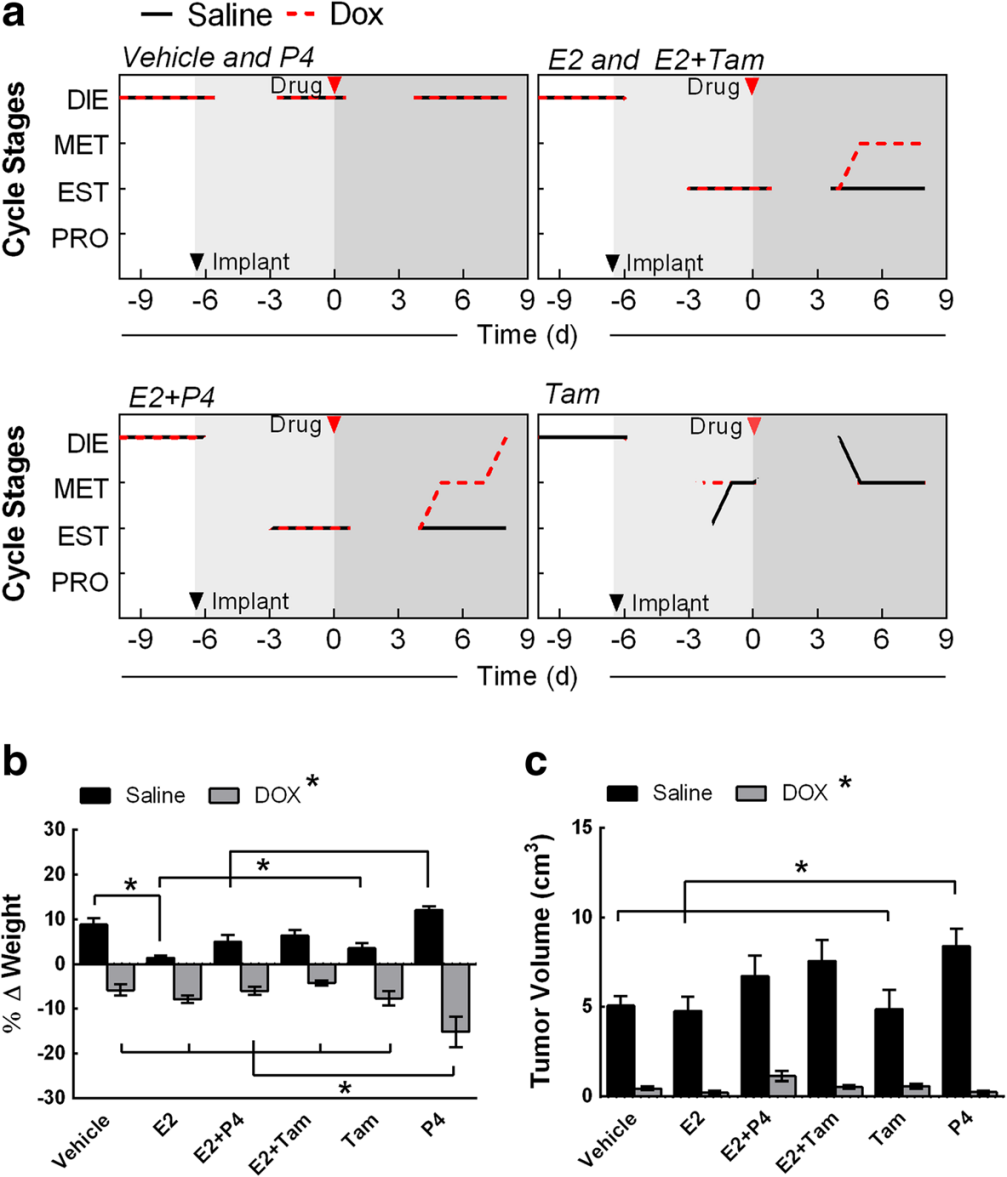
DOX suppresses exogenous 17β-estradiol- and progesterone-induced estrous cycle stimulation in ovariectomized SHRs. **a** Representative line graph depiction of the estrous cycle before any treatment for 3 days, after implantation at days 3 to 6 prior to DOX treatment, and post DOX treatment at days 3 to 8. Animals were implanted with time-release pellets that contain a control matrix (vehicle), 17β-estradiol (E2), progesterone (P4), tamoxifen (Tam), and combination treatments of E2 + Tam, and E2+ P4. DOX was administered 6 days following implantation. The estrous stages were determined by vaginal cytology in at least three animals per stage and treatment. Red arrow indicates implantation date and DOX treatment, respectively. **b**, **c** SST-2 tumor volume and weight of ovariectomized SHRs implanted with the time-release pellets at day 12 of a single saline, or DOX treatment (*n* = 6–7 per implant group per treatment). The bars represent the mean ± SEM. Two-way ANOVA results are displayed in Additional file 2: Table S3. *p < 0.05 according to Tukey’s multiple comparison between implants and treatment

To confirm DOX-induced estrous cycle irregularity in SHRs is partly dependent on circulating E2, we monitored the estrous cycle in the presence of hormonal treatments in E2-deficient ovaSHRs [18] before and after DOX administration. OvaSHRs were implanted with time-releasing pellets of E2, P4, and combinations of E2 with P4 and tamoxifen (Tam) then engrafted with SST-2 cells prior to DOX administration (Additional file 1: Figure S2). Using vaginal cytology, all ovaSHRs were determined to be in a prolonged diestrus (Fig. 4a). Supplementation with a vehicle or P4 did not stimulate the estrous cycle and the animals remained in prolonged diestrus in the presence and absence of DOX. Animals supplemented with E2 and combinations of E2 + P4 and E2 + Tam entered and remained in estrus in the absence of DOX. Administering DOX caused the E2 + P4 implanted rats to enter metestrus followed by diestrus on day 9, while E2 + Tam animals entered metestrus at day 5 post DOX treatment. In contrast to DOX, saline treatment did not cause transitions into other estrous stages in pellet-implanted SHRs, except for Tam-implanted animals. These data suggest that exogenous administration of E2 can affect the estrous cycle in ovaSHRs.

Tumor burden, weight, and uterine width were monitored for changes following time-released pellet implantation at day 12 post saline or DOX injection. Similar to previous reports [29, 32], uterine width increased in the presence of E2 but not P4 in SHRs (Additional file 1: Figure S7) (implant effect, *p* < 0.0001). The uterine width of ovaSHRs implanted with P4 remained comparable to the vehicle-implanted animals, which ranged from 0.10 to 0.12 cm. However, E2-implanted animals significantly increased the width of the uterus to 0.33 cm. After treatment with DOX, the uterine width was similar to saline-injected animals in ovaSHRs implanted with the vehicle (control) and P4 (0.09 to 0.14 cm), but DOX did cause a modest but significant decrease in the uterine width of E2-implanted ovaSHRs (0.28 cm) (treatment effect, *p* = 0.0167).

In the body weight assessment, all saline-treated ovaSHRs displayed a net increase in weight ranging from 1 to 12% at necropsy (Fig. 4b and Additional file 1: Figure S8a). E2-implanted ovaSHRs receiving a saline treatment did gain less weight than the vehicle-treated animals (1.4 to 8%) (interaction, *p* < 0.0001, implant and treatment). Animals that received implants containing P4 increased their initial body weight by 12%, which was greater than the E2-, E2 + P4- (5%), and Tam- (3.5%) implanted animals (Fig. 4b). DOX treatment caused a decline in the weight gain of all implanted animals, while P4-implanted animals had the greatest weight loss (− 15.5%). Tumor volume was also assessed every 2–4 days. A significant tumor developed in all saline-treated ovaSHRs and DOX suppressed tumor growth in all pellet-implanted ovaSHRs (treatment effect, *p* = < 0.0001). P4-implanted animals in the absence of DOX was the only group that had greater tumor burden as compared to vehicle-, E2- and Tam-implanted animals (interaction, *p* = 0.0484, implant and treatment). This indicates that tumor growth suppression was not dependent on hormone or Tam supplementation alone (5.0 to 8.8 cm^3^) (Fig. 4c and Additional file 1: Figure S8b). To further investigate the receptor status of SST-2 cells, immunoblot were performed to revealed that SST-2 cells were negative for estrogen receptors (ER), progesterone receptors (PGR), and human epidermal growth factor receptor (HER2) as compared to the MCF-7 cell line (ER^+^, PGR^+^, and HER2^−^) (Additional file 1: Figure S9). Collectively, these data demonstrate that DOX suppresses tumor growth during exogenous hormone supplementation in the SST-2/ovaSHRs model.

### DOX influences the circulating exogenous E2 and P4 bioavailability

**Fig. 5:**
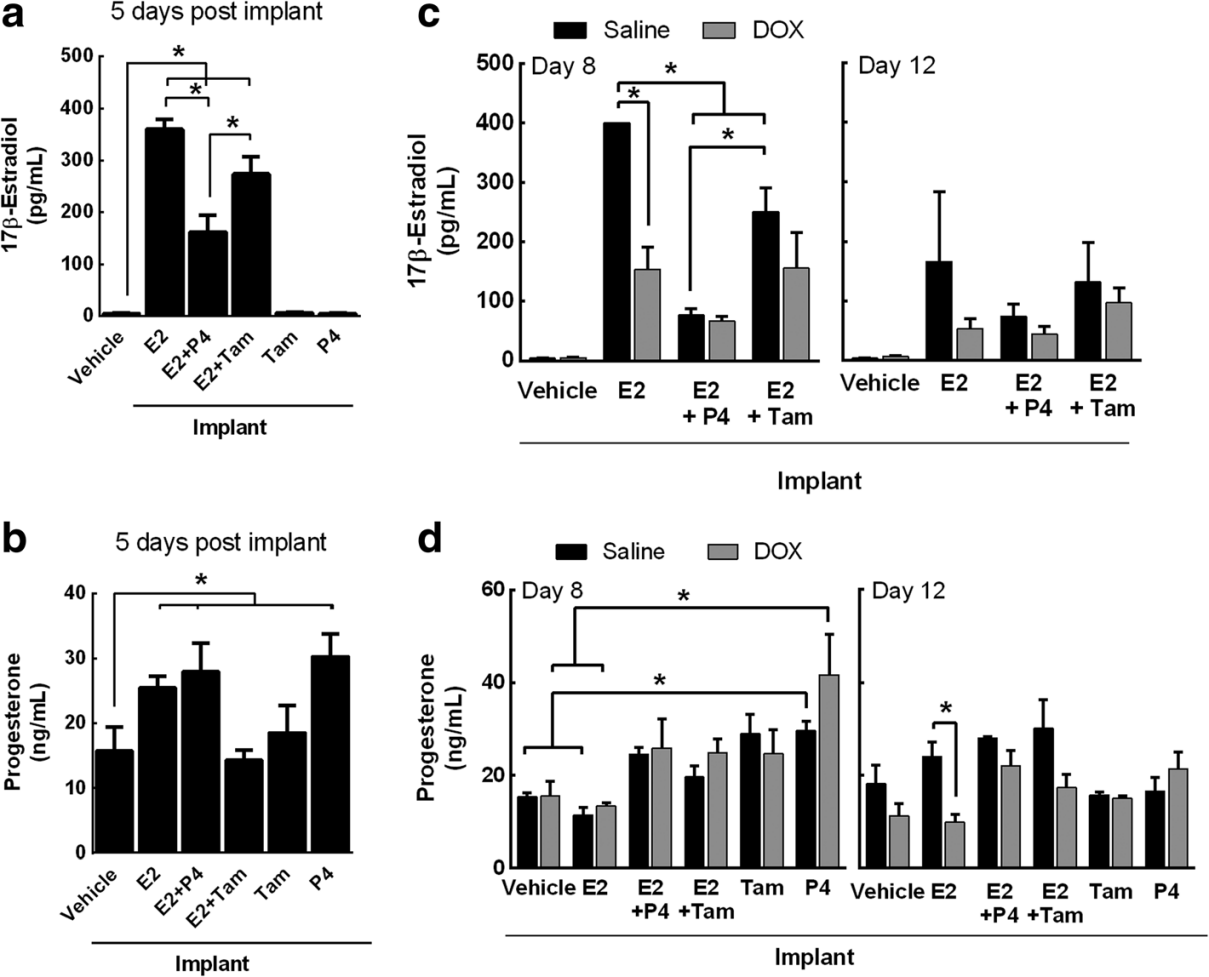
DOX affects the serum concentrations of E2 and P4 following exogenous administration in ovariectomized SHRs. **a**, **b** Serum concentrations of E2 and P4 were measured in ovariectomized SHRs implanted with no pellet (sham) or a pellet containing a vehicle matrix (control), E2, P4, Tam, and a combination of E2 + P4 and E2 + Tam at day 5, which is before any DOX treatment (ANOVA, *n* = 6–8 per implant, *p  <0.05 according to Dunnett’s multiple comparison between implants). **c**, **d** E2 and P4 concentrations were measured at days 8 and 12 after DOX treatment in pellet-implanted SHRs (two-ANOVA, *n* = 3–4 per implant per treatment, *p  <0.05 according to Tukey’s multiple comparison between implants and treatment). The bars represent the mean ± SEM

To investigate if DOX affects exogenous hormone bioavailability in ovaSHRs, the serum concentrations of E2 and P4 were quantified before and after a saline or DOX treatment from ovaSHRs that had been implanted with time-released pellets. At day 5 post hormone pellet implantation, serum concentrations of E2 were higher in E2- (360 pg/mL), E2 + P4- (161 pg/mL), and E2 + Tam- (274 pg/mL) implanted ovaSHRs as compared to vehicle-implanted animals (5 pg/mL) (*p* < 0.0001) (Fig. 5a). Exogenous E2 concentrations in the serum of ovaSHRs decreased when combined with P4 and Tam indicating that these combined therapies affect the bioavailability of exogenous E2 in the blood of ovaSHRs. As for serum progesterone concentrations, E2- (25 ng/mL), E2 + P4- (28 ng/mL), and P4- (29 ng/mL) implanted animals contained the greatest P4 levels as compared to the vehicle-implanted animals (16 ng/mL) (*p* = 0.0001) (Fig. 5b). Tam alone did not increase E2 or P4. These data indicate that the exogenous hormone pellet-implanted ovaSHRs increased circulating E2 and P4 levels, but combining E2 with P4 and Tam reduced the serum E2 concentration.

To establish the concentration of exogenous E2 and P4 in the circulation of ovaSHRs over time, serum from pellet-implanted animals was analyze for changes in the E2 and P4 concentration on days 8 and 12 after a saline injection. E2 concentrations in ovaSHRs that had received E2- (day 8, 400 pg/mL; day 12, 166 pg/mL), E2 + P4- (day 8, 76 pg/mL, day 12, 73 pg/mL), and E2 + Tam- (day 8, 250 pg/mL; day 12, 131 pg/mL) implants were greater than the vehicle-implanted ovaSHRs (day 8, 4 pg/mL; day 12, 4 pg/mL) (Fig. 5c) (implant effect, *p* < 0.0001). After 8 days, the saline-injected animals with E2-implants had maintained a similar serum E2 concentrations as compared to day 5 post implantation prior to DOX administration (repeat measure (time) ANOVA, *p* = 0.182). However, prolonging the study to 12 days led to a 130 pg/mL reduction in E2 concentrations in saline-injected ovaSHRs implanted with E2, which may suggest that E2 was being removed or degraded in these ovaSHRs. E2 + Tam-implanted ovaSHRs contained lower E2 concentrations following a saline injection, but displayed a similar E2 reduction trend between days 8 and 12. E2 + P4-implanted ovaSHRs exhibited an 80 pg/mL loss in E2 between day 5 post implantation to day 8 post DOX treatment, which maintained until day 12. These data further support that combining E2 with P4 can decrease the bioavailability of circulating exogenous E2 in ovaSHRs.

To identify how DOX affects the circulating exogenous E2 and P4 concentrations, serum from saline- and DOX-treated ovaSHRs implanted with E2, E2 + P4, and E2 + Tam was analyzed. At day 8 post injection, DOX treatment led to a 240 pg/mL loss of exogenous E2 circulating in the blood of E2-implanted ovaSHRs (interaction, *p* = 0.0007, implant and treatment) (Fig. 5c). OvaSHRs implanted with pellets that contained E2 with P4 or Tam maintained E2 levels after a DOX injection. At 12 days post DOX treatment, no differences between the groups or treatments were identified. This data suggests that exogenous E2 in the serum of E2-implanted ovaSHRs decreases in the presence of DOX.

As for the P4 concentrations over time in saline-injected ovaSHRs (Fig. 5d), the serum concentrations only modestly changed in animals that received a pellet containing E2 (day 8, 11 pg/mL; day 12, 24 pg/mL), E2 + P4 (day 8, 24 pg/mL; day 12, 28 pg/mL), and P4 (day 8, 30 pg/mL; day 12, 17 pg/mL) as compared to the vehicle-pelleted animals (day 8, 15 pg/mL; day 12, 18 pg/mL) (implant effect, *p* = 0.0003). P4-implanted ovaSHRs had a significant change in P4 levels in the presence and absence of DOX as compared to the vehicle- and E2-implanted animals at day 8 post treatment. At 12 days post injection, E2-implanted animals demonstrated a significant decreased progesterone levels in DOX-treated animals (treatment effect, *p* = 0.0035) (Fig. 5d). Collectively, these data show DOX affects circulating E2 and P4 levels in animals that received exogenous E2.

### Exogenous E2 suppresses DOX-induced cardiotoxicity in ovaSHRs

**Fig. 6:**
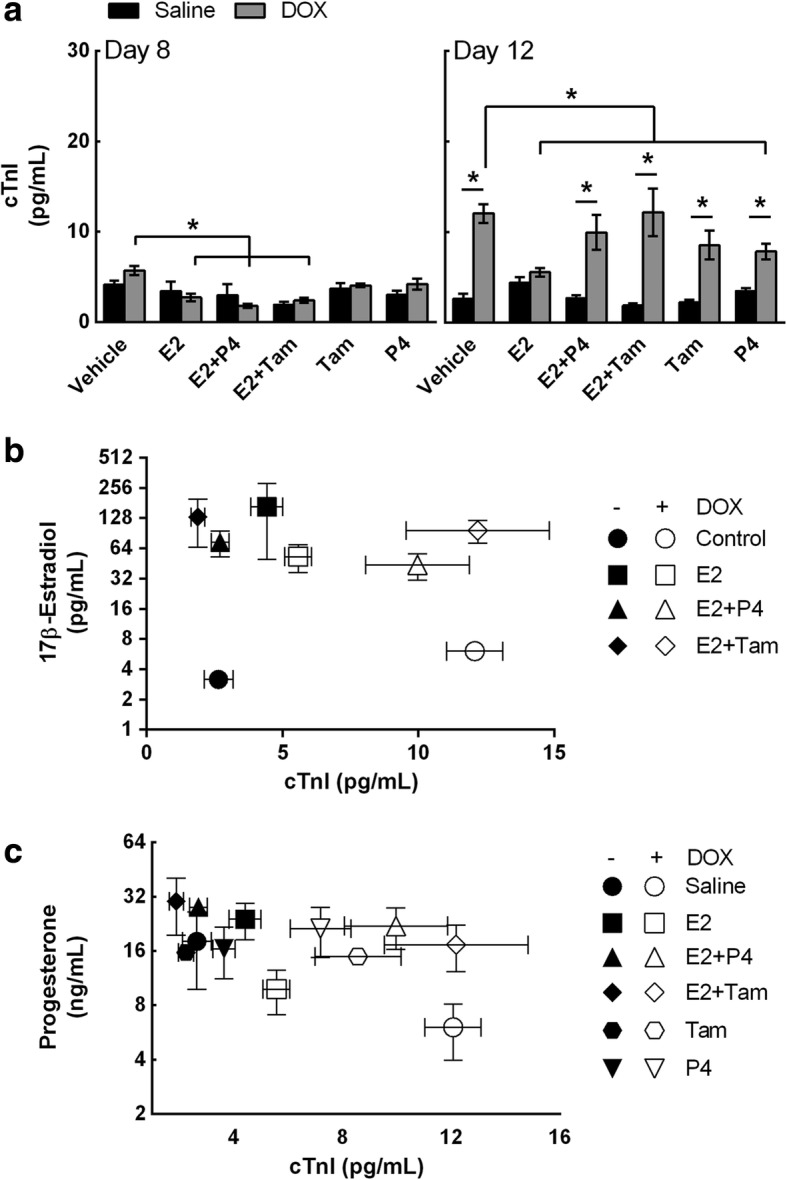
Exogenous 17β-estradiol and progesterone suppresses cardiac damage in ovariectomized SHR. **a** Serum concentrations of cardiac troponin I (cTnI) were measured from animals that contained pellet implants at days 8 and 12 post DOX treatment (two-way ANOVA, *n* = 4–6 per implant per treatment, *p  <0.05 according to Tukey’s multiple comparison between implants and treatment). Bars represent the mean ± SEM. **b**, **c** Scatter graph to evaluate the relationship between the levels of serum cardiac troponin I and serum E2 or P4 of pellet-implanted SHRs in the presence and absence of DOX treatment at day 12 (*n* = 3–4 per implant per treatment)

To investigate if exogenous E2 and P4 suppressed DOX-induced myocardial damage in SHRs, serum cTnI concentrations were measured from hormone pellet-implanted SHRs that were injected with saline or DOX (Fig. 6a). At day 8 post injection, cTnI levels modestly increased by 3 pg/mL in vehicle-implanted SHRs (4 pg/mL) treated with DOX indicating the potential onset of cardiac damage. Prolonging the time to 12 days caused a more significant increase in cTnI levels (14 pg/mL) in the DOX-treated ovaSHRs as compared to the control (4 pg/mL) (treatment effect, *p* < 0.0001). In the hormone pellet-implanted ovaSHRs, DOX did not cause a significant increase in cTnI at day 8 post treatment. However, ovaSHRs that received E2 (day 8, 2 pg/mL) alone or in combination with P4 (day 8, 2 pg/mL) or Tam (day 8, 2 pg/mL) exhibited lower cTnI values as compared to the vehicle-implanted animals treated with DOX (implant effect, *p* = 0.0015). Extending the post treatment time to 12 days led to a significant increase in the serum cTnI levels of DOX-treated ovaSHRs implanted with E2 + P4, E2 + Tam, and Tam alone that ranged between 8 pg/mL and 14 pg/mL. E2-implanted animals were the only group to have a comparable serum cTnI (4 pg/mL) concentration in the presence and absence of DOX (interaction, *p* = 0.0028, implant and treatment). P4-implanted SHRs did appear to at least partly suppress the increase in cTnI levels by 6 pg/mL as compared to the DOX-treated control animals, but the levels were still greater than the saline-injected animals with P4 implants.

To further investigate myocardial damage, histopathological cardiac tissue sections were assessed to identify cardiomyocyte lesions (Table 2 and Additional file 1: Figure S10). No cardiac lesions were observed in saline-treated animals, independent of hormone/Tam addition. However, DOX treatment caused moderate cardiac lesions and the severity was associated with hormone/Tam supplementation (Table 2). DOX-treated vehicle (1.9), Tam (2.0), and Tam + E2 (1.8) all displayed lesion scores (LS) greater than 1.5, where P4 (1.3), E2 (1.4), and E2 + P4 (1.2) all displayed LS less than 1.5 (Table 2). Ultimately, minor LS were associated with low cTnI values with the exception of DOX-treated ovaSHRs with E2 + P4 implants. Moreover, given the high LS and cTnI values, the addition of Tam completely negated any cardioprotective effect E2 may have had, which suggests this selective estrogen receptor modulator (SERM) interferes with or inhibits E2-mediated cardioprotection from DOX (Fig. 6a and Table 2). Overall these results show that exogenous E2 may at least partially protect against DOX-induced cardiomyopathy.

**Table 2:** OvaSHR cardiomyopathy score

Hormone	Treatment	n	0	1	1.5	2	2.5	Average
Vehicle	Saline	6	6					0.0
	DOX	7			4	1	2	1.9
E2	Saline	7	7					0.0
	DOX	7		3	2	2		1.4
E2 + P4	Saline	7	7					0.0
	DOX	7	1	2	3	1		1.2
E2 + Tam	Saline	7	7					0.0
	DOX	7			4	2	1	1.8
Tam	Saline	7	7					0.0
	DOX	7			3	1	3	2.0
P4	Saline	7	7					0.0
	DOX	7	1	1	4	1		1.3

Histopathology of cardiac sections from the ovariectomized SHRs at day 12 was assessed for cardiomyocyte lesions (myofibrillar loss and vacuolization) by a board-certified pathologist. The cardiomyopathy or lesion score indicates the percentage of affected cardiomyocytes within the sampled heart section: 0 is no observed change, 1 is < 5%, 1.5 is 5–15%, 2 is 16–25%, 2.5 is 26–35%, and 3 is > 35%

To establish a relationship between cardiac damage and reproductive hormones, the cTnI concentrations were compared to the E2 or P4 concentrations using scatter plot analysis. These data suggest a relationship may exist between E2 and cTnI in E2-, E2 + Tam-, and E2 + P4-implanted ovaSHRs (Fig. 6b). In the presence of DOX, but in the absence of any supplement (vehicle), the serum cTnI was high and serum E2 was low supporting an inverse relationship between E2 and cTnI that has been reported previously [18]. E2 combined with P4 or Tam caused an increase in the serum cTnI levels in the presence of DOX suggesting that P4 and Tam may suppress E2-mediated cardioprotection from DOX. OvaSHRs that received P4 therapy either alone or in combination exhibited moderate progesterone levels and cTnI values (Fig. 6c). Collectively, these data suggest that exogenous E2 suppresses DOX-induced myocardial damage in ovaSHRs.

## Discussion

These findings provide the first evidence that demonstrates that estrous stage and exogenous hormones can affect DOX-induced cardiac damage in two tumor-bearing female adult SHR models. In this report, the SST-2/SHR model was utilized to investigate DOX-induced estrous cycle irregularities, tumor growth and cardiac damage, while a SST-2/ovaSHR model was used to investigate exogenous E2-mediated cytoprotection against DOX-induced cardiotoxicity. Here, we demonstrate that estrous-staged DOX treatments induced estrous cycle irregularity but had no impact on the efficacy of DOX as a chemotherapeutic in tumor-bearing SHRs. Acute estrous cycle irregularity (< 9 days), the duration until the onset of prolonged diestrus, and the extent of cardiac damage were in part dependent on the estrous-staged DOX treatment (Figs. 1 and 2). This could imply that the endogenous hormones present during each stage of the estrous cycle may contribute to the level of DOX-induced cardiotoxicity. Second, we compared the E2 and P4 levels to cTnI to identify if the endogenous hormones can affect DOX-induced cardiotoxicity (Figs. 2 and 3). Proestrus contained the highest levels of E2, but administering DOX caused the greatest myocardial damage. Third, exogenous E2 can suppress DOX-induced cardiotoxicity in ovaSHRs (Figs. 5 and 6). E2 supplementation in the presence of DOX suppressed an increase in serum cTnI levels, which may indicate that E2 is cardioprotective agent against DOX in ovaSHRs. Collectively, this study shows that these preclinical tumor-bearing SHR models can be useful research tools to study DOX-induced estrous cycle irregularity and E2-induced cardioprotection against DOX.

DOX-induced cardiotoxicity is an adverse effect for patients that received chemotherapy [41]. Dexrazoxane is the only approved drug for DOX-induced cardiotoxicity for breast cancer patients with a cumulative dose of 300 mg/m^2^ or a continuous DOX treatment. Consistent with our previous study [18], SHRs have an increase in cTnI levels and cardiac lesion scores in the presence of DOX and DRZ. This indicates that DRZ did not abolish DOX-induced cardiotoxicity. This contradicted Herman et al.’s finding that demonstrates DRZ suppressed DOX-induced cardiotoxicity in the female SHRs using cardiac lesion scores [42]. In Herman et al.’s study, weekly injections of DOX and DRZ were performed for 12 weeks. In our studies, a single dose of DOX and DRZ was administered, then followed for a maximum of 14 days, which may be a limitation in this study.

Here, we demonstrate that cTnI levels do not increase in the serum of DOX-treated SHRs until day 12 post treatment. One can speculate that this may imply that at an earlier time point, such as day 6 post treatment, the extent of cardiac damage did not lead to cardiomyocyte death. cTnI has been used as an indicator for the onset of cardiac damage [43], but less is known about the correlation between serum cTnI levels and the extent of cardiac damage. Here, we show that proestrus-staged SHRs treated with DOX have an increase in cTnI levels and a significant decline in cardiac output as compared to estrus- and diestrus-staged animals that received DOX. Furthermore, proestrus-staged SHRs treated with DRZ in combination with DOX suppressed the elevation in serum cTnI levels and increased cardiac output. However, cardiac lesion scores that evaluate the tissue structure could not differentiate between these treatments. This indicates the estrous cycle may cause variation in ability of DRZ to suppress DOX-induced cardiotoxicity at early time points and that proestrus may enhance the sensitivity of cardiac tissue to DOX, which both require further investigation.

Premenopausal women undergo fluctuations in E2 and P4 during the menstrual cycle [38, 40]. Exogenous administration of E2 and P4 has demonstrated to provide cytoprotection against several different acute and chronic myocardial stressors [17, 22, 26, 27, 44, 45]. The role of endogenous hormones providing cytoprotection against DOX-induced myocardial dysfunction is unknown. Consistent with other reports [28, 35, 36, 39], we show that proestrus has the highest E2 levels in the SHR model. However, DOX treatment during proestrus led to heightened cardiac damage as compared to DOX treated SHRs during estrus, metestrus, and diestrus. This indicates that heightened endogenous E2 levels did not partly suppress DOX-induced cardiotoxicity in SHRs. SHRs are known to already possess cardiac damage prior to DOX treatment [27, 42, 46]; therefore, a non-hypertensive rat model should be used for further investigations. Collectively, our study provides the first evidence that DOX treatment during a specific estrous stage can influence the extent of DOX-induced cardiotoxicity.

Administration of exogenous E2 is an experimental strategy that has been shown to ameliorate DOX-induced cardiotoxicity in male and females Wistar rats [12, 17]. Here, we show that ovariectomized female SHRs administered E2 suppressed DOX-induced cardiotoxicity. OvaSHR-administered E2 have demonstrated to suppress oxidative stress and improve left ventricular reactivity [26]. Hypertensive rat cardiac tissue does contain heightened NAD(P)H oxidase activity and superoxide production as compared to aged-match Wistar rats [46]. Reactive oxygen species have been proposed to contribute to DOX-induced cardiotoxicity [47]. Utilizing this ovaSHR model, the oxidative state of the cardiac tissue will be further analyzed to identify the role of exogenous E2 in modulating the redox homeostasis of rat cardiac tissue following DOX treatment. This may provide further insight into the mechanism that contributes to E2 suppressing DOX-induced cardiotoxicity in SHRs.

Chemotherapy-induced amenorrhea is a side effect of DOX treatment in premenopausal women [48]. The rate of breast cancer in women younger than 40 is increasing [49], but our understanding of how DOX affects the reproductive system in limited. Female Wistar rats administered DOX have demonstrated to undergo reproductive toxicity [39]. Here, we demonstrate, using vaginal cytology, that the DOX induces estrous cycle irregularity leading to prolonged diestrus in a normal reproductive hypertensive rat. This preclinical model may be useful to investigate the effects DOX on the reproductive system in SHRs for young women with hypertension that received DOX.

In 2002, The Women’s Health Initiative (WHI) study found that the use of estrogen and progesterone therapy increased the risk for heart disease and breast cancer in post-menopausal women [45, 50]. Since this observational study, significant controversy has persisted over the drug formulation, time and mode of delivery, and genetic variation between subjects [45, 51–53]. Here, this data demonstrates that slow-release hormone pellets that contain 17β-estradiol quench DOX-induced cardiotoxicity in ovariectomized hypertensive female rats. The cardioprotection conveyed by E2 against DOX was suppressed by progesterone indicating that progesterone may suppress the E2 cardioprotective effects. This can be further supported by progesterone ability to antagonize the effects of E2 in the aorta of rats administered noradrenalin [54]. Furthermore, exogenous P4 enhanced tumor growth and did not provide protection against DOX-induced cardiotoxicity in ovaSHRs. The increase in tumor volume was an unexpected result because SST-2 cells were identified as a triple negative cell line (estrogen receptor (ER)-negative, progesterone receptor-negative, and HER2-negative). These findings and utilizing this model may contribute to understanding the unanticipated negative effects of the WHI hormone therapy study on post-menopausal women.

## Conclusions

In this study, we demonstrated that estrous cycling SHRs in proestrus are more sensitive to DOX-induced cardiotoxicity as compared to the other estrous stages, and 17β-estradiol suppresses DOX-induced cardiomyopathy in ovaSHRs. Collectively, we have established two preclinical hypertensive rat models to investigate (1) DOX-induced reproductive cycle irregularity and (2) 17β-estradiol-induced protection from DOX-induced cardiotoxicity. Utilizing these models, the risk and severity of adverse events in hypertensive pre- and post-menopausal women can be assessed prior to clinical trials.

## Additional files

Additional file 1: Study design and supporting material. **Figure S1**. SHR study design to evaluate estrous cycle effects on DOX-induced cardiotoxicity. **Figure S2**. OvaSHR study design to evaluate the effects of exogenous hormone supplementation and Dox-induced cardiotoxicity. **Figure S3**. DRZ does not affect estrous cycle in SHRs. **Figure S4**. Uterine width changes in SHRs. **Figure S5**. DRZ does not affect the weight, tumor growth or uterine width in SHRs. **Figure S6**. DRZ alone does not cause cardiotoxicity in SHRs. **Figure S7**. Uterine width changes in ovaSHRs administered exogenous E2 and P4. **Figure S8**. Weight and tumor volume changes in hormone-implanted ovaSHRs. **Figure S9**. Hormone receptor expression in MCF-7 and SST-2 cell lines. (PDF 1172 kb)

Additional file 2: Procedure group sizes and statistical analyses. **Table S1**. Treatment groups and group sizes for procedures and investigational analyses using spontaneous hypertensive rat model. **Table S2**. Treatment groups and group sizes for procedures and investigational analyses using ovariectomized spontaneous hypertensive rat model. **Table S3**. Statistical results of the two-way ANOVA analyses for the spontaneous hypertensive rats (SHR) and ovariectomized spontaneous hypertensive rats (ovaSHR) models. **Table S4**. Statistical results of the two-way ANOVA analyses with time as a repeated measure for the spontaneous hypertensive rat (SHR) model. **Table S5**. Statistical results of the one-way ANOVA analyses for the spontaneous hypertensive rat (SHR) and ovariectomized spontaneous hypertensive rat (ovaSHR) models. **Table S6**. Statistical results of the one-way ANOVA analyses with time as a repeated measure for the spontaneous hypertensive rat (SHR) and ovariectomized spontaneous hypertensive rat (ovaSHR) models. (DOCX 28 kb)

## Abbreviations

cTnI: Cardiac troponin I
DIE: Diestrus
DOX: Doxorubicin
DRZ: Dexrazoxane
E2: 17β-Estradiol
ERα or ERβ: Estrogen receptor alpha or beta
EST: Estrus
H&E: Hematoxylin and eosin
IV: Intravenous
MET: Metestrus
ovaSHRs: Ovariectomized spontaneous hypertensive rats
P4: Progesterone
PGR: Progesterone receptor
PRO: Proestrus
SHRs: Spontaneously hypertensive rats
SST-2: Syngeneic breast tumor cells from SHR
WHI: Women’s Health Initiative
