# Supplementary material for: Doxorubicin-induced cardiotoxicity is suppressed by estrous-staged treatment and exogenous 17β-estradiol in female tumor-bearing spontaneously hypertensive rats
Source: Biol Sex Differ. 2018 Jun 15;9:25. doi: 10.1186/s13293-018-0183-9 (PMC6003183; doi:10.1186/s13293-018-0183-9)
Supplement: Supplementary file 1 — Study design and supporting material. Figure S1. SHR study design to evaluate estrous cycle effects on DOX-induced cardiotoxicity. Figure S2. OvaSHR study design to evaluate the effects of exogenous hormone supplementation and Dox-induced cardiotoxicity. Figure S3. DRZ does not affect estrous cycle in SHRs. Figure S4. Uterine width changes in SHRs. Figure S5. DRZ does not affect the weight, tumor growth or uterine width in SHRs. Figure S6. DRZ alone does not cause cardiotoxicity in SHRs. Figure S7. Uterine width changes in ovaSHRs administered exogenous E2 and P4. Figure S8. Weight and tumor volume changes in hormone-implanted ovaSHRs. Figure S9. Hormone receptor expression in MCF-7 and SST-2 cell lines. (PDF 1172 kb) [file 13293_2018_183_MOESM1_ESM.pdf]

Fig S1: SHR study design to evaluate estrous cycle affects on DOX-induced cardiotoxicity.

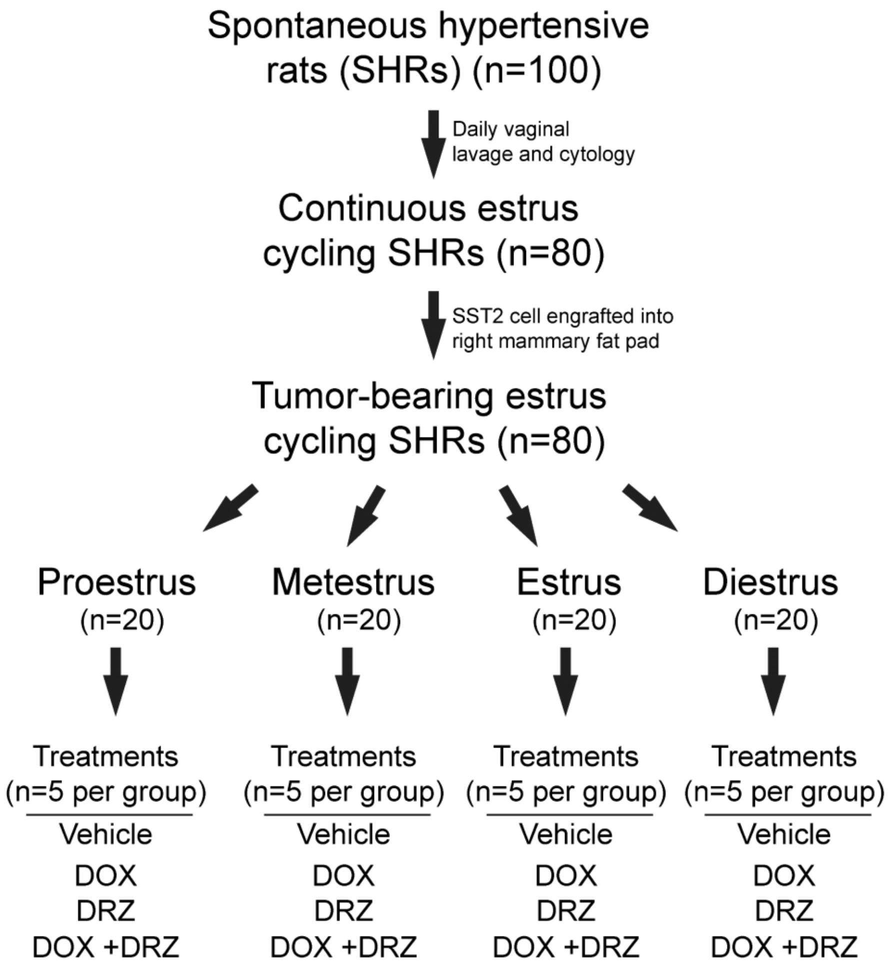

Flow chart that depicts the estrus-staged SHRs study. SHRs (n=100) were received and allowed to acclimate for 35 days. Prior to the start of the study, SHRs underwent vaginal lavage and cytology to identify two consecutive regular estrous cycles in 80 rats. These SHRs then subcutaneously engrafted with 8.5 x 10^6 SST-2 cells into the right mammary fat pad. After 24 hours, the animals were divided to cohorts based on the estrous stage (proestrus, estrus, metestrus or diestrus) (n=20) and treated once with injectable saline (0.9%) or DOX (10 mg/kg) via intravenous (IV) lateral tail vein injection, the cardioprotectant Dexrazoxane (DRZ; 50 mg/kg) via intraperitoneal injection (IP) or the combination of both DOX+DRZ (n=5 per treatment).

Fig S2: OvaSHR study design to evaluate the affects of exogenous hormone supplementation and Dox-induced cardiotoxicity.

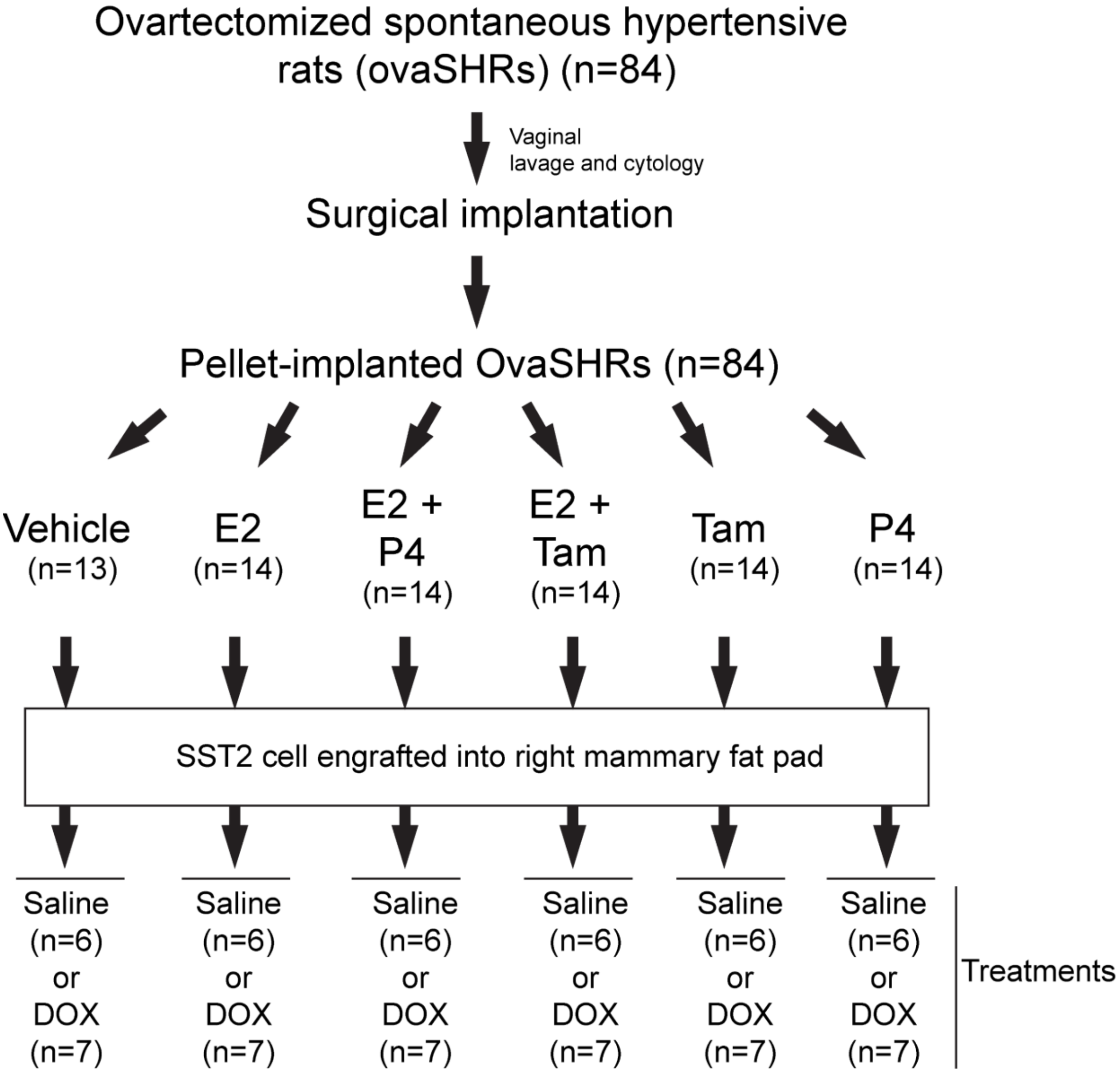

Flow chart that depicts the ovariectomized SHRs (ovaSHRs) study. OvaSHRs (n=84) were acclimated for 5 days. During the last 3 days vaginal lavage and cytology was performed. The ovaSHRs then underwent surgery to implant time-release pellets. Pellet implants contained a carrier matrix (vehicle) and E2, E2 + P4, E2 + Tam, Tam or P4 (n=13-14 per implant). The animals were acclimatized to the implants for 6 days. The SHRs then subcutaneously implanted with of 8.5 x 10^6 SST-2 cells into the right mammary fat pad. At 24 hours post engraftation, animals were treated with saline or DOX (n=6-7 per treatment).

Fig S3: DRZ does not affect estrous cycle in SHRs.

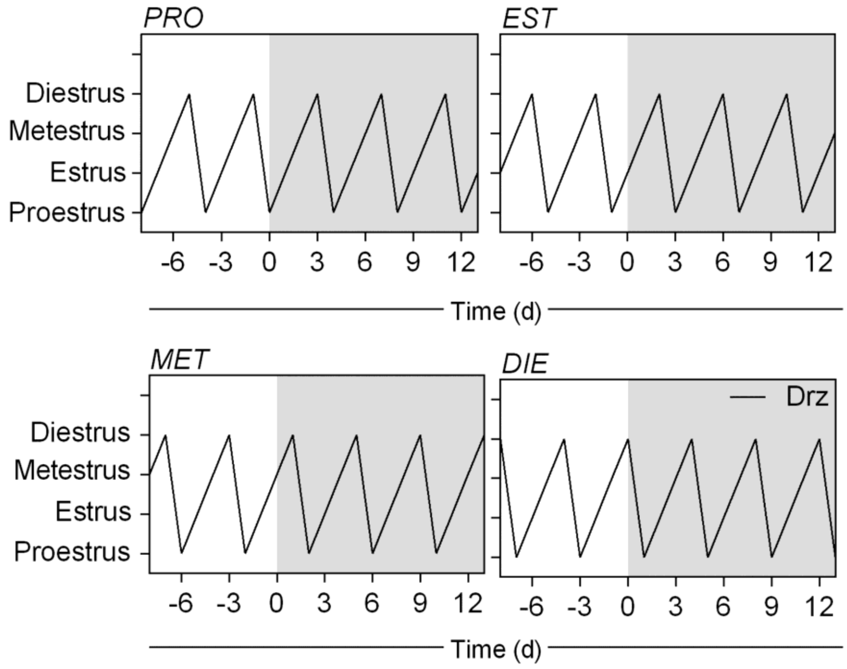

DRZ treatment alone did not affect the estrous cycle in SHRs. Representative line graph depiction of the estrous cycle for 13 days following a single injection of saline or DRZ during PRO, EST, MET, or DIE. The estrous stages were determined by vaginal cytology in at least 3 animals per stage and treatment.

Fig S4: Uterine width changes in SHRs.

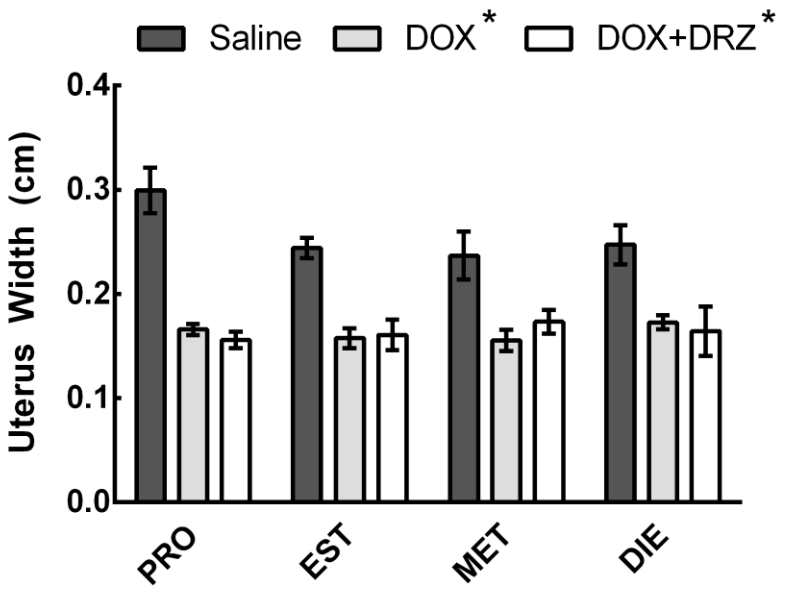

Uterus width of SHRs following DOX treatment. Uterus width changes in SHRs treated with DOX at day 12. The bars represent the mean ± SEM. (Two-way ANOVA, *p < 0.05 according to Dunnett's multiple comparison between the treatments within the stage.)

Fig S5: DRZ does not affect the weight, tumor growth or uterine width in SHRs.

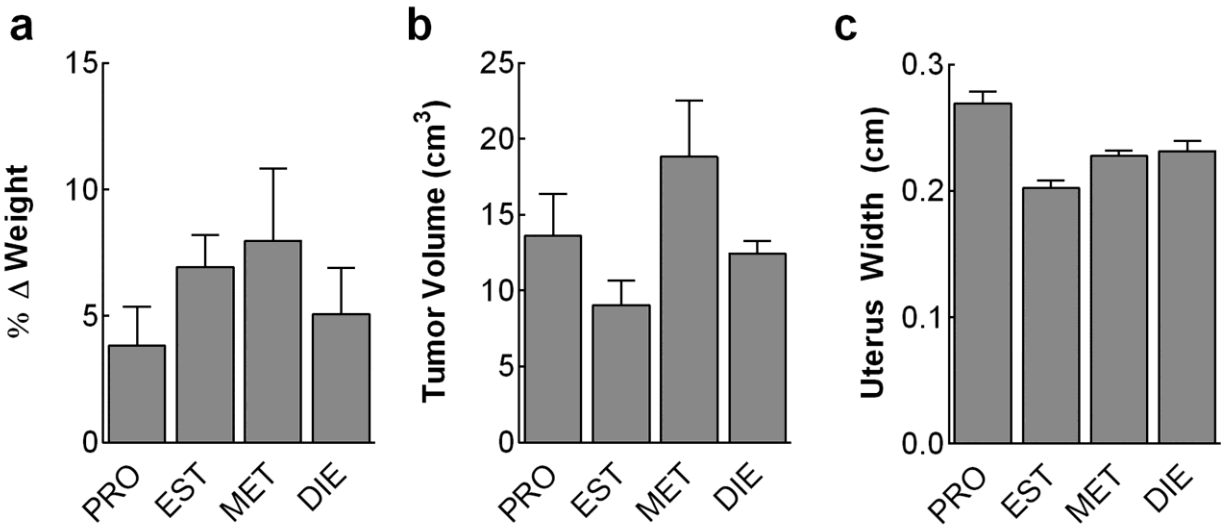

DRZ treatment alone did not affect SHR weight, tumor growth, uterus width, and cardiac damage. a, **b**, **c** Weight, tumor volume, and uterus width following DRZ treatment at day 13 in estrous stage specific SHRs (n=4 per stage). Weight, tumor volume, and uterine width in SHRs treated with DRZ was not statistically different than saline-treated SHRs. Two-way ANOVA results are listed in Additional file2: Table S3.

Fig S6: DRZ alone does not cause cardiotoxicity in SHRs.

a

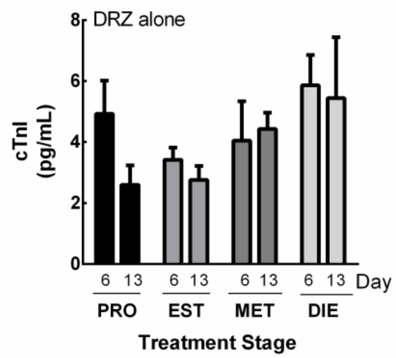

b

Cardiomyopathy Score								
Stage	Treatment	n	0	1	2	3	4	Avg
DIE	Drz	5	2	3				0.6
PRO	Drz	5	1	4				0.8
EST	Drz	5	2	3				0.6
MET	Drz	5	1	4				0.8

c

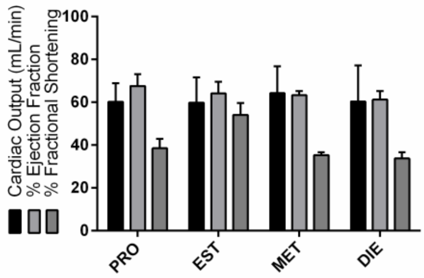

DRZ alone did not lead to myocardial dysfunction or damage. **a** Serum concentrations of cardiac troponin I (cTnI) were measured from animals that received estrous stage specific DRZ treatment at day13 (n= 3 per stage). **b** Cardiomyopathy score of histopathologic cardiac sections from the SHRs treated with DRZ at day 12. **c** Echocardiograms were performed 13 days post DRZ treatment in estrous stage specific SHRs. Cardiac output, % ejection fraction, and % fractional shortening were analyzed to assess myocardial dysfunction (n=3 per stage). Two-way ANOVA results are listed in Additional file2: Table S3.

Fig S7: Uterine width changes in ovaSHRs administered exogenous E2 and P4.

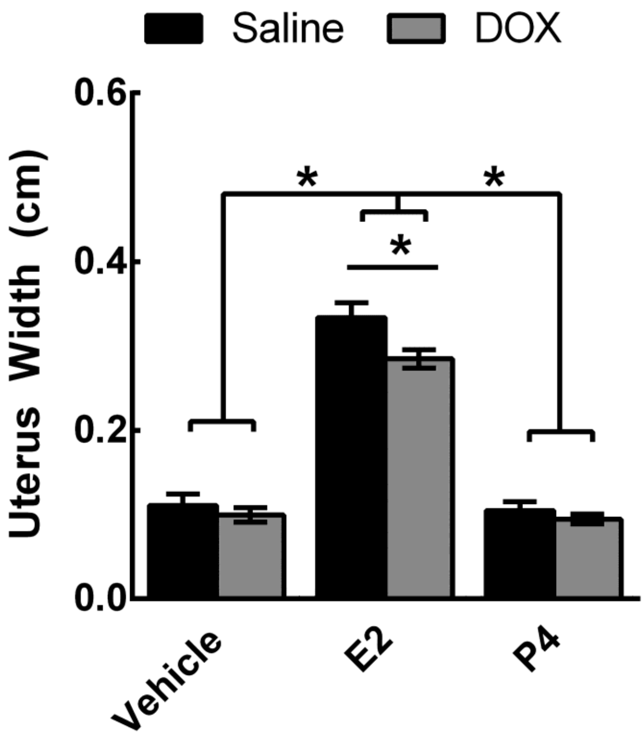

Uterine width size increases with exogenous E2 administration. The uterus width was measured in vehicle, E2 and P4 pellet-implanted SHRs at day 12 (Two-way ANOVA, n=3 per group, *$p<0.05$ according to Sidak's multiple comparison between treatment and implants.). The bars represent the mean ± SEM. *

Fig S8: Weight and tumor volume changes in hormone implanted ovaSHRs.

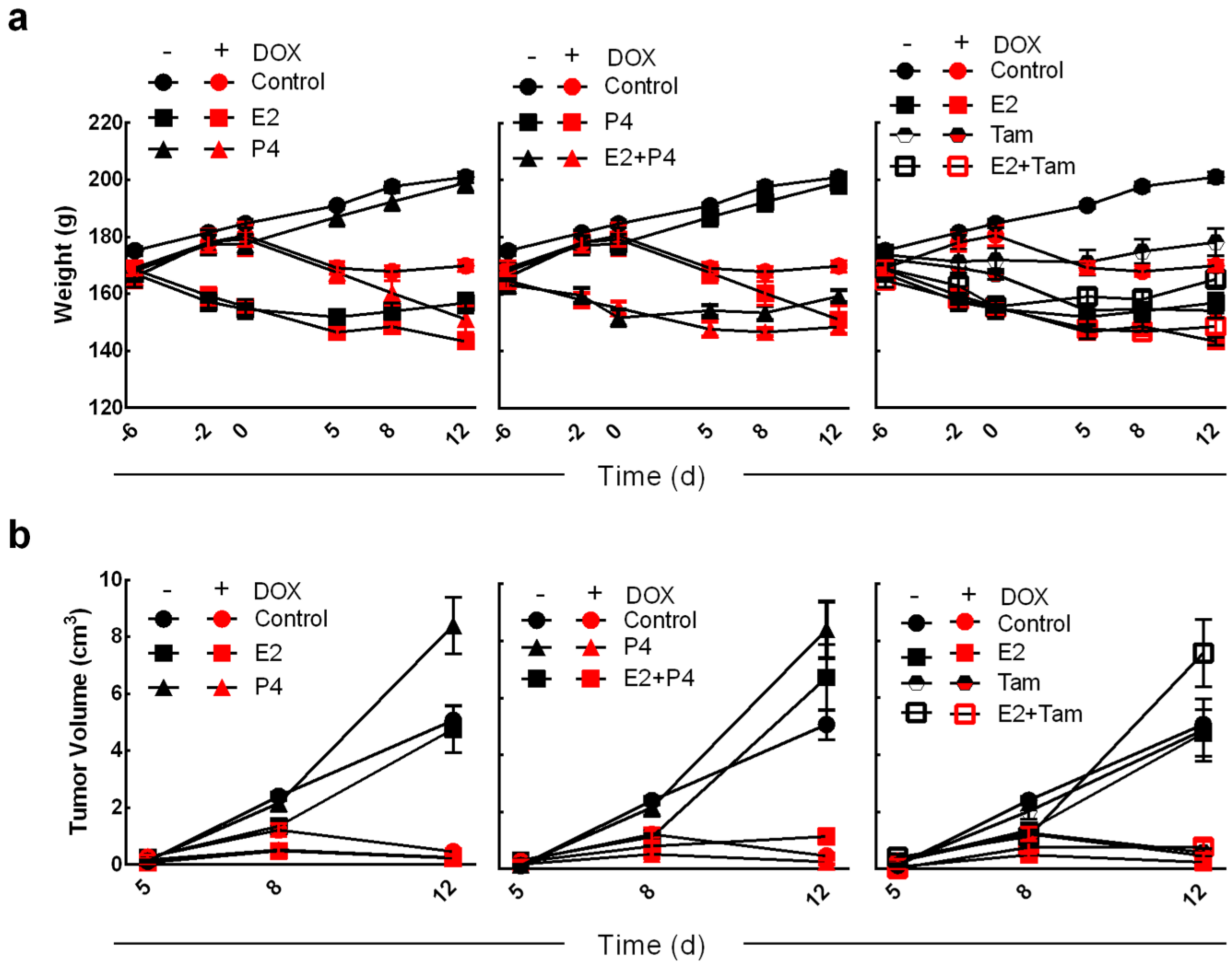

Weight and tumor volume of ovariectomized SHRs implanted with a time-release hormone pellet. **a** The weight of the pellet-implanted SHRs was measured before and after DOX administration at the times indicated (n=4 per implant per treatment). **b** The tumor volume was measured 5, 8 and 12 days following DOX treatment in pellet-implanted SHRs (n=4 per implant per treatment). The dots represent the mean ± SEM.

Fig S9: Hormone receptor expression in MCF-7 and SST-2 cell lines.

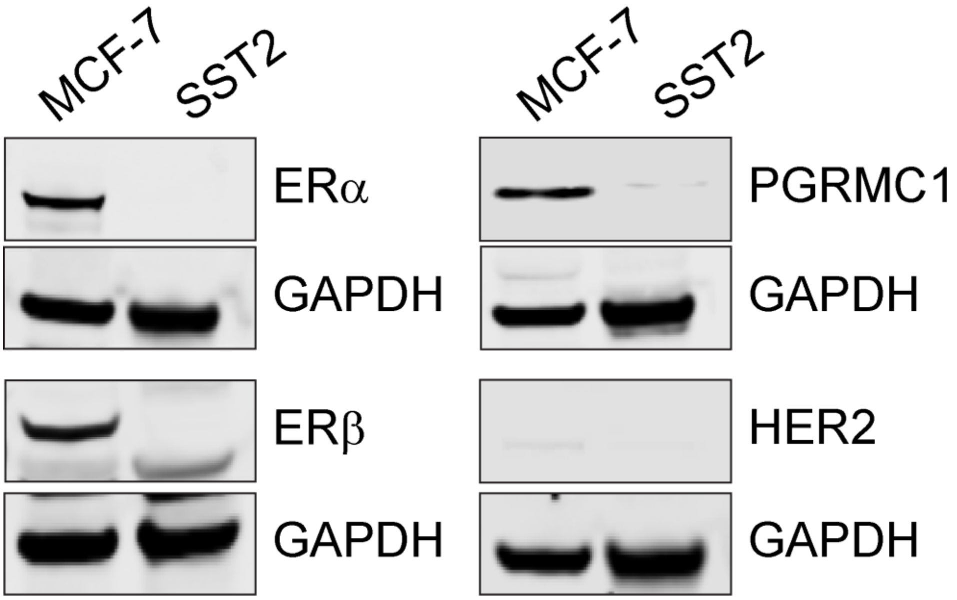

SST2 cells are a rat mammary triple negative cell line. Representative immunoblots of estrogen receptor α (ERα), estrogen receptor β (ERβ), human epidermal growth factor receptor (HER2), and progesterone receptor membrane component 1 (PGRMC1) in MCF7 and SST2 cells.

Fig S10: Cardiac histopathology sections from pellet-implanted ovaSHRs.

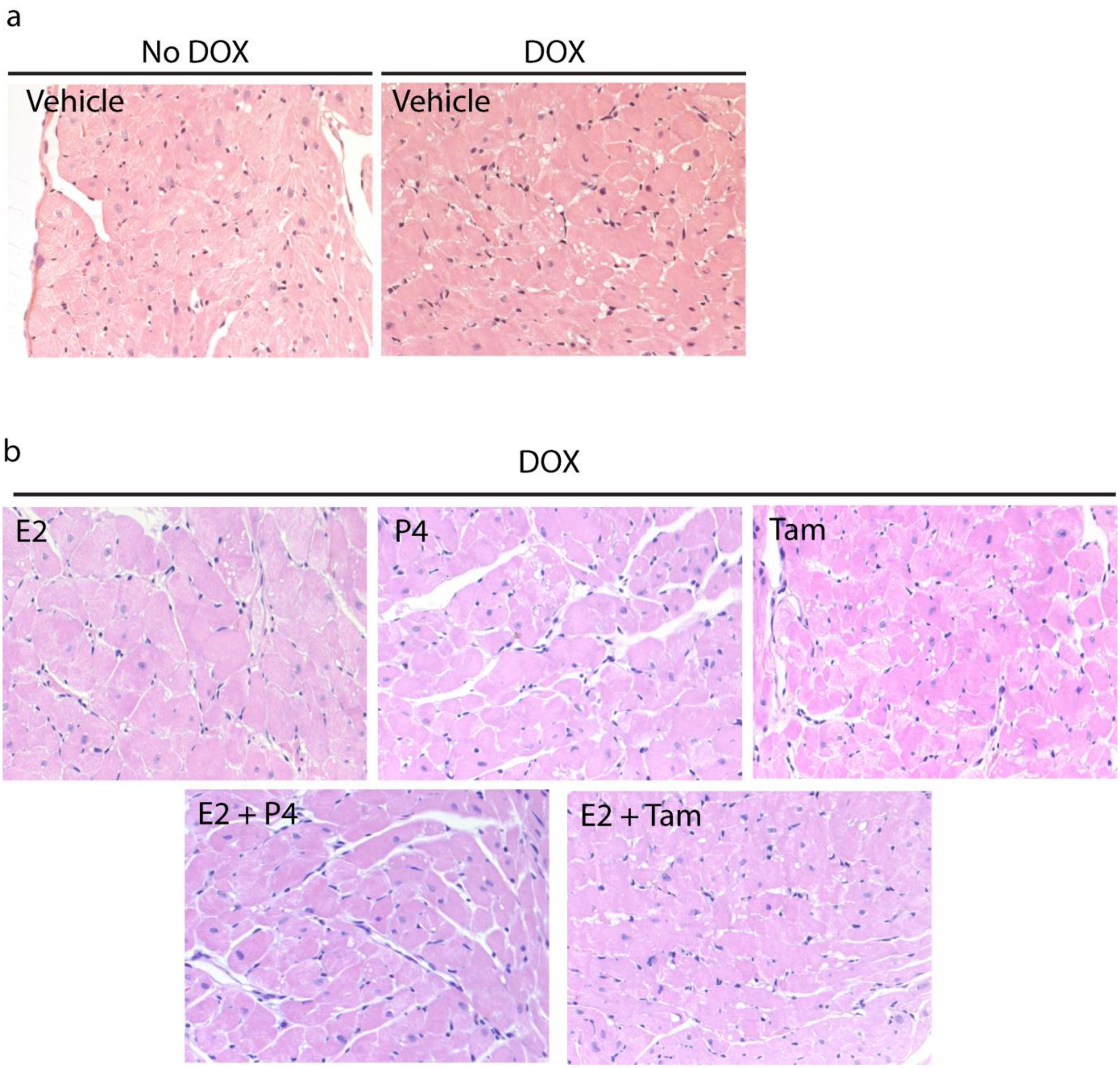

Hematoxylin and eosin stained cardiac sections from pellet-implanted ovaSHRs. a Representative images of cardiac tissue from rats implanted with vehicle (control) containing pellets in the presence and absence of DOX treatment. **b** Representative images of cardiac tissue from DOX-treated rats implanted with pellets containing E2, P4, Tam or combinations of E2 with P4 and Tam.
