# Supplementary material for: Doxorubicin-induced cardiotoxicity is suppressed by estrous-staged treatment and exogenous 17β-estradiol in female tumor-bearing spontaneously hypertensive rats
Source: Biol Sex Differ. 2018 Jun 15;9:25. doi: 10.1186/s13293-018-0183-9 (PMC6003183; doi:10.1186/s13293-018-0183-9)
Supplement: Supplementary file 2 — Procedure group sizes and statistical analyses. Table S1. Treatment groups and group sizes for procedures and investigational analyses using spontaneous hypertensive rat model. Table S2. Treatment groups and group sizes for procedures and investigational analyses using ovariectomized spontaneous hypertensive rat model. Table S3. Statistical results of the two-way ANOVA analyses for the spontaneous hypertensive rats (SHR) and ovariectomized spontaneous hypertensive rats (ovaSHR) models. Table S4. Statistical results of the two-way ANOVA analyses with time as a repeated measure for the spontaneous hypertensive rat (SHR) model. Table S5. Statistical results of the one-way ANOVA analyses for the spontaneous hypertensive rat (SHR) and ovariectomized spontaneous hypertensive rat (ovaSHR) models. Table S6. Statistical results of the one-way ANOVA analyses with time as a repeated measure for the spontaneous hypertensive rat (SHR) and ovariectomized spontaneous hypertensive rat (ovaSHR) models. (DOCX 28 kb) [file 13293_2018_183_MOESM2_ESM.docx]

| Spontaneous Hypertensive Rats | | | | | | | | | |
| --- | --- | --- | --- | --- | --- | --- | --- | --- | --- |
|  |  | Procedure/Investigational Analyses (group size) | | | | | | | |
| Groups | Treatment | Weight | Tumor  burden | Uterine  length | Vaginal  lavage/cytology | Echocardography | Cardiac  Troponin | Histopathology | E2 and  P4 levels |
| Proestrus | Vehicle | 5 | 5 | 5 | 5 | 3 | 3 | 5 | 3 |
|  | DOX | 5 | 5 | 5 | 5 | 3 | 3 | 5 | 3 |
|  | DRZ | 5 | 5 | 5 | 5 | 3 | 3 | 5 | NP |
|  | DOX+DRZ | 5 | 5 | 4 | 5 | 3 | 3 | 5 | 3 |
| Estrus | Vehicle | 5 | 5 | 5 | 5 | 3 | 3 | 5 | 3 |
|  | DOX | 5 | 5 | 5 | 5 | 3 | 3 | 5 | 3 |
|  | DRZ | 5 | 5 | 5 | 5 | 3 | 3 | 5 | NP |
|  | DOX+DRZ | 5 | 5 | 5 | 5 | 3 | 3 | 5 | 3 |
| Metestrus | Vehicle | 4 | 4 | 4 | 4 | 3 | 3 | 5 | 3 |
|  | DOX | 4 | 4 | 4 | 4 | 3 | 3 | 5 | 3 |
|  | DRZ | 4 | 4 | 4 | 5 | 3 | 3 | 5 | NP |
|  | DOX+DRZ | 4 | 4 | 4 | 4 | 3 | 3 | 5 | 3 |
| Diestrus | Vehicle | 5 | 5 | 5 | 5 | 3 | 3 | 5 | 3 |
|  | DOX | 5 | 5 | 4 | 5 | 3 | 3 | 5 | 3 |
|  | DRZ | 5 | 5 | 5 | 5 | 3 | 3 | 5 | NP |
|  | DOX+DRZ | 5 | 5 | 4 | 5 | 3 | 3 | 5 | 3 |

Table S1: Treatment groups, and group sizes for procedures and investigational analyses perform using spontaneous hypertensive rat model.

Table S1: Spontaneous hypertensive rats were divided in 4 groups based on the estrus phrase: proestrus, estrus, metestrus, and diestrus. Each animal was administered a vehicle (control), DOX, DRZ, or DOX + DRZ. The groups and the group sizes for each procedure and investigational analyses can be located within this table. NP is not performed.

Table S2: Treatment groups, and group sizes for procedures and investigational analyses perform using ovariectomized spontaneous hypertensive rat model.

| Ovariectomized Spontaneous Hypertensive Rats | | | | | | | | |
| --- | --- | --- | --- | --- | --- | --- | --- | --- |
|  |  | Procedure/Investigational Analyses (group size) | | | | | | |
| Pellet  implanted | Injection | Weight | Tumor  burden | Uterine  length | Vaginal  lavage/cytology | Cardiac  Troponin | Histopathology | E2 and P4 levels |
| Vehicle | Saline | 7 | 6 | 4 | 7 | 5 | 6 | 4 |
|  | DOX | 7 | 6 | 5 | 7 | 5 | 7 | 4 |
| E2 | Saline | 7 | 6 | 5 | 7 | 6 | 7 | 3 |
|  | DOX | 7 | 6 | 6 | 7 | 6 | 7 | 3 |
| E2 + P4 | Saline | 7 | 6 | NP | 7 | 5 | 7 | 3 |
|  | DOX | 7 | 6 | NP | 7 | 5 | 7 | 3 |
| E2 + Tam | Saline | 7 | 6 | NP | 7 | 5 | 7 | 3 |
|  | DOX | 7 | 6 | NP | 7 | 5 | 7 | 3 |
| Tam | Saline | 7 | 6 | NP | 7 | 5 | 7 | 3 |
|  | DOX | 7 | 6 | NP | 7 | 5 | 7 | 3 |
| P4 | Saline | 7 | 6 | 6 | 7 | 6 | 7 | 3 |
|  | DOX | 7 | 6 | 6 | 7 | 6 | 7 | 3 |

Table S2: Ovariectomized spontaneous hypertensive rats were implanted with different time releasing hormone pellets followed by an injection with saline or DOX. The groups and the group sizes for each procedure and investigational analyses can be located within this table. NP is not performed.

Table S3: Statistical results of the two-way ANOVA analyses for the spontaneous hypertensive rats (SHR) and ovariectomized spontaneous hypertensive rats (ovaSHR) models.

| **Two-Way ANOVA** | | | | | | |
| --- | --- | --- | --- | --- | --- | --- |
| **Model** | **Parameter** | **Location** | **Effect** | **DF** | **F (DFn,DFd)** | **P Value** |
| SHR | Tumor Growth | Figure 1c &  Additional File1:  Fig S5b | Interaction | 9 | F (9,57) = 0.9970 | P =0.4531 |
|  |  |  | Estrous Stage | 3 | F (3,57) = 1.571 | P = 0.2054 |
|  |  |  | Treatment | 3 | F (3,57) = 60.86 | P < 0.0001 |
|  | SHR Weight change | Figure 1d &  Additional File1:  Fig S5a | Interaction | 9 | F (9,59) = 3.183 | P = 0.0034 |
|  |  |  | Estrous Stage | 3 | F (3,59) = 3.586 | P = 0.0189 |
|  |  |  | Treatment | 3 | F (3,59) = 46.45 | P < 0.0001 |
|  | Uterine Width | Additional File1:  Fig S4 & S5c | Interaction | 9 | F (9, 56) = 3.185 | P = 0.0035 |
|  |  |  | Estrous Stage | 3 | F (3, 56) = 7.059 | P = 0.0004 |
|  |  |  | Treatment | 3 | F (3, 56) = 65.84 | P < 0.0001 |
|  | Cardiac output | Figure 2a | Interaction | 6 | F (6, 24) = 2.873 | P = 0.0296 |
|  |  |  | Estrous Stage | 3 | F (3, 24) = 1.087 | P = 0.3734 |
|  |  |  | Treatment | 2 | F (2, 24) = 11.05 | P = 0.0004 |
|  | Ejection Fraction | Figure 2a | Interaction | 6 | F (6, 24) = 1.723 | P = 0.1588 |
|  |  |  | Estrous Stage | 3 | F (3, 24) = 3.400 | P = 0.0340 |
|  |  |  | Treatment | 2 | F (2, 24) = 7.009 | P = 0.0040 |
|  | Fraction Shortening | Figure 2a | Interaction | 6 | F (6, 24) = 1.723 | P = 0.1968 |
|  |  |  | Estrous Stage | 3 | F (3, 24) = 3.400 | P <0.0001 |
|  |  |  | Treatment | 2 | F (2, 24) = 7.009 | P = 0.0097 |
|  | Cardiac output | Additional File1:  Fig S6c | Interaction | 3 | F (3, 16) = 1.441 | P = 0.2678 |
|  |  |  | Estrous Stage | 3 | F (3, 16) = 0.5639 | P = 0.6466 |
|  |  |  | Treatment | 1 | F (1, 16) = 0.07268 | P = 0.7909 |
|  | Ejection Fraction | Additional File1:  Fig S6c | Interaction | 3 | F (3, 16) = 2.626 | P = 0.0860 |
|  |  |  | Estrous Stage | 3 | F (3, 16) = 4.765 | P = 0.0147 |
|  |  |  | Treatment | 1 | F (1, 16) = 1.490 | P = 0.2399 |
|  | Fraction Shortening | Additional File1:  Fig S6c | Interaction | 3 | F (3, 16) = 0.5224 | P = 0.6730 |
|  |  |  | Estrous Stage | 3 | F (3, 16) = 26.80 | P < 0.0001 |
|  |  |  | Treatment | 1 | F (1, 16) = 5.332 | P = 0.0346 |
|  | cTNI Day 6 | Figure 2b &  Additional File1:  Fig S6a | Interaction | 9 | F (9, 35) = 0.6549 | P = 0.7427 |
|  |  |  | Estrous Stage | 3 | F (3, 35) = 0.8245 | P = 0.4893 |
|  |  |  | Treatment | 3 | F (3, 35) = 7.039 | P = 0.0008 |
|  | cTNI Day 13 | Figure 2b &  Additional File1:  Fig S6a | Interaction | 9 | F (9, 42) = 1.100 | P = 0.3669 |
|  |  |  | Estrous Stage | 3 | F (3, 42) = 1.430 | P = 0.2117 |
|  |  |  | Treatment | 3 | F (3, 42) = 27.95 | P < 0.0001 |
|  | 17beta-Estradiol  Day 6 | Figure 3b | Interaction | 6 | F (6, 24) = 1.457 | P = 0.0007 |
|  |  |  | Estrous Stage | 3 | F (3, 24) = 3.993 | P = 0.0194 |
|  |  |  | Treatment | 2 | F (2, 24) = 3.235 | P = 0.0934 |
|  | 17beta-Estradiol  Day 13 | Figure 3b | Interaction | 6 | F (6, 24) = 1.820 | P = 0.1376 |
|  |  |  | Estrous Stage | 3 | F (3, 24) = 3.143 | P = 0.0438 |
|  |  |  | Treatment | 2 | F (2, 24) = 6.541 | P = 0.0054 |
|  | Progesterone  Day 6 | Figure 3d | Interaction | 6 | F (6, 26) = 0.2916 | P = 0.9355 |
|  |  |  | Estrous Stage | 3 | F (3, 26) = 1.454 | P = 0.2501 |
|  |  |  | Treatment | 2 | F (2, 26) = 3.447 | P = 0.0470 |
|  | Progesterone  Day 13 | Figure 3d | Interaction | 6 | F (6, 25) = 1.415 | P = 0.2483 |
|  |  |  | Estrous Stage | 3 | F (3, 25) = 1.473 | P = 0.2460 |
|  |  |  | Treatment | 2 | F (2, 25) = 1.471 | P = 0.2489 |
| OvaSHR | SHR Weight change | Figure 4b | Interaction | 5 | F (5, 71) = 10.37 | P < 0.0001 |
|  |  |  | Implant | 5 | F (5, 71) = 3.031 | P = 0.0155 |
|  |  |  | Treatment | 1 | F (1, 71) = 266.7 | P < 0.0001 |
|  | Tumor Growth | Figure 4c | Interaction | 5 | F (5, 70) = 2.365 | P = 0.0484 |
|  |  |  | Implant | 5 | F (5, 70) = 2.497 | P = 0.0387 |
|  |  |  | Treatment | 1 | F (1, 70) = 185.7 | P < 0.0001 |
|  | Uterine Width | Additional File1:  Fig S7 | Interaction | 2 | F (2, 29) = 1.941 | P = 0.1617 |
|  |  |  | Implant | 2 | F (2, 29) = 226.1 | P < 0.0001 |
|  |  |  | Treatment | 1 | F (1, 29) = 6.458 | P = 0.0167 |
|  | 17beta-Estradiol  8 days post DOX | Figure 5c | Interaction | 3 | F (3, 18) = 9.031 | P = 0.0007 |
|  |  |  | Implant | 3 | F (3, 18) = 42.07 | P < 0.0001 |
|  |  |  | Treatment | 1 | F (1, 18) = 21.28 | P = 0.0002 |
|  | 17beta-Estradiol  12 days post DOX | Figure 5c | Interaction | 3 | F (3, 18) = 0.5959 | P = 0.6258 |
|  |  |  | Implant | 3 | F (3, 18) = 2.586 | P = 0.0850 |
|  |  |  | Treatment | 1 | F (1, 18) = 1.902 | P = 0.1848 |
|  | Progesterone  8 days post DOX | Figure 5d | Interaction | 5 | F (5, 26) = 1.018 | P = 0.4273 |
|  |  |  | Implant | 5 | F (5, 26) = 9.328 | P < 0.0001 |
|  |  |  | Treatment | 1 | F (1, 26) = 1.499 | P = 0.2318 |
|  | Progesterone 12 days post DOX | Figure 5d | Interaction | 5 | F (5, 26) = 2.486 | P = 0.0573 |
|  |  |  | Implant | 5 | F (5, 26) = 3.695 | P = 0.0117 |
|  |  |  | Treatment | 1 | F (1, 26) = 10.29 | P = 0.0035 |
|  | cTnI  8 days post DOX | Figure 6a | Interaction | 5 | F (5, 40) = 1.320 | P = 0.2751 |
|  |  |  | Implant | 5 | F (5, 40) = 4.835 | P = 0.0015 |
|  |  |  | Treatment | 1 | F (1, 40) = 0.4387 | P = 0.5115 |
|  | cTnI 12 days post DOX | Figure 6a | Interaction | 5 | F (5, 46) = 4.269 | P = 0.0028 |
|  |  |  | Implant | 5 | F (5, 46) = 1.241 | P = 0.3058 |
|  |  |  | Treatment | 1 | F (1, 46) = 77.78 | P < 0.0001 |

Table S3: Listed are the parameters, results, and figure location for all analyses that used a two-way ANOVA statistical analysis.

Table S4: Statistical results of the two-way ANOVA analyses with time as a repeated measure for the spontaneous hypertensive rats (SHR) model.

| **Two-way ANOVA with time as a repeated measure** | | | | | | |
| --- | --- | --- | --- | --- | --- | --- |
| **Model** | **Parameter** | **Location** | **Effect** | **DF** | **F (DFn,DFd)** | **P Value** |
| SHR | Proestrus-staged Day 1, 8, and 12 | Figure 1e | Interaction | 4 | F (4, 24) = 13.25 | P < 0.0001 |
|  |  |  | Treatment | 2 | F (2, 12) = 22.35 | P < 0.0001 |
|  |  |  | Time | 2 | F (2, 24) = 33.26 | P < 0.0001 |
|  |  |  | Subjects | 12 | F (12, 24) = 10.44 | P < 0.0001 |
|  | Estrus-staged  Day 1, 8 and 12 | Figure 1e | Interaction | 4 | F (4, 24) = 11.25 | P < 0.0001 |
|  |  |  | Treatment | 2 | F (2, 12) = 7.791 | P = 0.0068 |
|  |  |  | Time | 2 | F (2, 24) = 29.21 | P < 0.0001 |
|  |  |  | Subjects | 12 | F (12, 24) = 10.04 | P < 0.0001 |
|  | Metestrus-staged  Day 1, 8, and 12 | Figure 1e | Interaction | 4 | F (4, 18) = 9.387 | P = 0.0003 |
|  |  |  | Treatment | 2 | F (2, 9) = 3.464 | P = 0.0766 |
|  |  |  | Time | 2 | F (2, 18) = 40.14 | P < 0.0001 |
|  |  |  | Subjects | 9 | F (9, 18) = 6.221 | P = 0.0005 |
|  | Metestrus-staged  Day 1, 8, and 12 | Figure 1e | Interaction | 4 | F (4, 24) = 13.25 | P < 0.0001 |
|  |  |  | Treatment | 2 | F (2, 12) = 22.35 | P < 0.0001 |
|  |  |  | Time | 2 | F (2, 24) = 33.26 | P < 0.0001 |
|  |  |  | Subjects | 12 | F (12, 24) = 10.44 | P < 0.0001 |

Table S4: Listed are the parameters, results, and figure location for all analyses that used a two-way ANOVA statistical analysis with time as a repeated measure.

Table S5: Statistical results of the one-way ANOVA analyses for the spontaneous hypertensive rats (SHR) and ovariectomized spontaneous hypertensive rats (ovaSHR) models.

| **Ordinary one-way ANOVA** | | | | | | |
| --- | --- | --- | --- | --- | --- | --- |
| **Model** | **Parameter** | **Location** | **Effect** | **DF** | **F (DFn,DFd)** | **P Value** |
| SHR | 17beta-Estradiol | Figure 3a | Stage | 3 | F (3, 44) = 12.06 | P < 0.0001 |
|  | Progesterone | Figure 3b | Stage | 3 | F (3, 44) = 1.365 | P = 0.2658 |
| OvaSHR | 17beta-Estradiol | Figure 5a | Implant | 5 | F (5, 30) = 58.28 | P < 0.0001 |
|  | Progesterone | Figure 5 b | Implant | 5 | F (5, 30) = 6.693 | P = 0.0003 |

Table S5: Listed are the parameters, results, and figure location for all analyses that used a one-way ANOVA statistical analysis.

Table S6: Statistical results of the one-way ANOVA analyses with time as a repeated measure for the spontaneous hypertensive rats (SHR) and ovariectomized spontaneous hypertensive rats (ovaSHR) models.

| **One way ANOVA with repeated measure (time)** | | | | | |
| --- | --- | --- | --- | --- | --- |
| **Parameter** | **Location** | **Effect** | **DF** | **F (DFn,DFd)** | **P Value** |
| 17beta Estradiol clearance | Not shown | Time points  (5 days post implant, 8 and 12 Days post DOX) | 2 | F (2, 4) = 1.376 | P = 0.3509 |
| Progesterone  clearance | No Shown | Time points  (5 days post implant, 8 and 12 Days post DOX) | 2 | F (2, 4) = 4.875 | P = 0.0466 |

Table S6: Listed are the parameters, results, and figure location for all analyses that used a one-way ANOVA statistical analysis with time as a repeated measure.
